# Complete biosynthesis of salicylic acid from phenylalanine in plants

**DOI:** 10.1038/s41586-025-09175-9

**Published:** 2025-07-23

**Authors:** Bao Zhu, Yanjun Zhang, Rong Gao, Zhihua Wu, Wei Zhang, Chao Zhang, Penghong Zhang, Can Ye, Linbo Yao, Ying Jin, Hui Mao, Peiyao Tou, Peng Huang, Jiangzhe Zhao, Qiao Zhao, Chang-Jun Liu, Kewei Zhang

**Affiliations:** 1 Zhejiang Provincial Key Laboratory of Biotechnology on Specialty Economic Plants, College of Life Sciences, Zhejiang Normal University, Jinhua, China; 2 China-Mozambique “Belt and Road” Joint Laboratory on Smart Agriculture, Zhejiang Normal University, Jinhua, China; 3 College of Chemistry and Materials Science, Zhejiang Normal University, Jinhua, China; 4 Shenzhen Key Laboratory of Synthetic Genomics, Guangdong Provincial Key Laboratory of Synthetic Genomics, Key Laboratory of Quantitative Synthetic Biology, Shenzhen Institute of Synthetic Biology, Shenzhen Institute of Advanced Technology, Chinese Academy of Sciences, Shenzhen, China; 5 Biology Department, Brookhaven National Laboratory, Upton, NY USA

**Keywords:** Secondary metabolism, Plant immunity, Plant hormones

## Abstract

Salicylic acid (SA) is a pivotal phytohormone for plant responses to biotic and abiotic stresses. Plants have evolved two pathways to produce SA: the isochorismate synthase and phenylalanine ammonia lyase (PAL) pathways^1^. Whereas the isochorismate synthase pathway has been fully identified^2–4^, the PAL pathway remains incomplete. Here we report the full characterization of the PAL pathway for SA biosynthesis via functional analysis of rice (*Oryza sativa*) *SA-DEFICIENT GENE 1* (*OSD1*) to *OSD4*. The cinnamoyl-coenzyme A (CoA) ligase OSD1 catalyses the conversion of *trans*-cinnamic acid to cinnamoyl-CoA, which is subsequently transformed to benzoyl-CoA via the β-oxidative pathway in peroxisomes. The resulting benzoyl-CoA is further converted to benzyl benzoate by the peroxisomal benzoyltransferase OSD2. Benzyl benzoate is subsequently hydroxylated to benzyl salicylate by the endoplasmic reticulum membrane-resident cytochrome P450 OSD3, which is ultimately hydrolysed to salicylic acid by the cytoplasmic carboxylesterase OSD4. Evolutionary analyses reveal that the PAL pathway was first assembled before the divergence of gymnosperms and has been conserved in most seed plants. Activation of the PAL pathway in rice significantly enhances salicylic acid levels and plant immunity. Completion of the PAL pathway provides critical insights into the primary salicylic acid biosynthetic pathway across plant species and offers a precise target for modulating crop immunity.

## Abstract (web summary)

Characterization of four SALICYLIC ACID-DEFICIENT GENEs in rice identifies an ancestral pathway for biosynthesis of salicylic acid from phenylalanine and provides genetic targets for engineering disease resistance in crop plants.

## Main

Salicylic acid, a natural phenolic, has been studied for its medicinal use in humans for more than 200 years^5^, and has emerged as a phytohormone in biotic and abiotic stress responses in the past three decades. Plants synthesize SA via two independent metabolic pathways, the isochorismate synthase and PAL pathways^1,6,7^. The isochorismate synthase pathway has been studied in various plant species and was fully established in the dicotyledonous model plant *Arabidopsis thaliana*^2–4,8–14^. The PAL pathway has long been known to contribute to SA biosynthesis in many plant species^6,7,11,14–24^. Isotope-labelling experiments demonstrated that SA can be synthesized from phenylalanine (Phe) via *trans*-cinnamic acid (*trans*-CA) and benzoic acid (BA) in tobacco (*Nicotiana tabacum*)^22^. The β-oxidation pathway, comprising enzymes such as cinnamate-CoA ligase (CNL), cinnamoyl-CoA hydratase/dehydrogenase (CHD) and 3-ketoacyl-CoA thiolase (KAT), has been shown to convert *trans*-CA to benzoyl-CoA (BA-CoA) and subsequently to BA in several plant species, including *Petunia hybrida*, *Hydpercum calycinum*, *Nicotiana benthamiana*, tobacco, *Populus trichocarpa* and *O. sativa*^23–30^. More recently, this pathway has been linked to SA biosynthesis in rice, tobacco and *N. benthamiana*, mediated by enzymes such as *Os*CNL and ABNORMAL INFLORESCENCE MERISTEM1 (AIM1) (also known as *Os*CHD) in rice, and the *Nt*CNL–*Nt*CHD–*Nt*KAT module in tobacco^23,27–30^. Additionally, a peroxisome-localized benzyl alcohol *O*-benzoyltransferase (BEBT), HSR201, has been found to be essential for pathogen signal-induced SA biosynthesis in tobacco and *N. benthamiana*^27,30,31^. Despite these advances, the complete phenylalanine-derived SA biosynthetic pathway remains unresolved. Notably, more than three decades ago, a presumptive benzoic acid 2-hydroxylase (BA2H), a soluble P450 enzyme, was proposed to catalyse the conversion of BA to SA in tobacco^32^. However, the molecular identity of BA2H has yet to be elucidated.

Rice, a staple crop for more than half of the world’s population and a monocotyledonous model plant, accumulates high basal levels of SA. Its SA concentration is ten times higher than that of *A. thaliana* and does not significantly increase upon pathogen exposure^33,34^. In rice, the PAL pathway, rather than the isochorismate synthase pathway, is responsible for basal SA biosynthesis^10,14,35^. Here we performed forward genetic screening for the mutants of *OSD1* and characterized the gene by map-based cloning and bulk population sequencing. We then identified and characterized three additional SA-deficient genes, *OSD2*, *OSD3* and *OSD4*, via gene co-expression analysis with *OSD1,* thereby establishing the complete PAL pathway from *trans*-CA to SA via BA-CoA as an intermediate. Through evolutionary analysis of the identified key enzymes involved in SA biosynthesis, we revealed that the PAL pathway for SA biosynthesis (PAL-SA pathway) emerged before the divergence of gymnosperms and has been conserved in most seed plants. Activation of the PAL-SA pathway in rice strongly increases SA content and plant immunity. Our study describes the full PAL-SA biosynthetic pathway, its cellular compartmentation and evolution, and offers a strategy for modulating plant immunity.

## Characterization of SA-deficient mutants

**Fig. 1:**
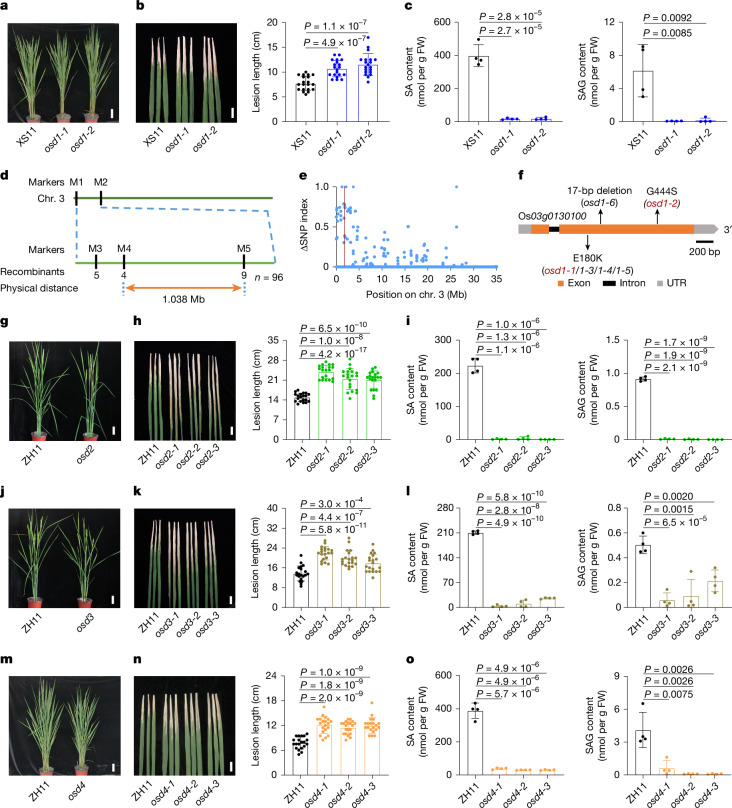
Characterization of the key genes required for SA biosynthesis in rice. **a**, Phenotypes of XS11 (Xiushui 11), *osd1-1* and *osd1-2* plants at the heading stage. Scale bar, 10 cm. **b**, Disease symptoms and lesion lengths measured at 14 days post-inoculation (dpi) of *Xoo*. Scale bar, 3 cm. **c**, The SA and SAG contents in the leaves of 14-day-old XS11, *osd1-1* and *osd1-2* plants. FW, Fresh weight. **d**, Map-based cloning of the *OSD1* gene. The gene was mapped to a 1.038-Mb genomic region by molecular markers M4 and M5 with 96 F_2_ recessive mutant lines. **e**, Bulk population sequencing of the locus underlying *osd1-1*. ΔSNP index of chromosome 3 between the two DNA pools is shown. Blue dots indicate positions exhibiting ΔSNP index values. The red line indicates the position of *osd1-1*. **f**, Gene structure of *OSD1*. The black arrow indicates the mutation position in *osd1-1*, *1-2*, *1–3*, *1–4*, *1–5* and *1–6*. Scale bar, 200 bp. **g**,**j**,**m**, Phenotypes of the wild-type, *osd2* (**g**), *osd3* (**j**) and *osd4* (**m**) plants at the heading stage. Scale bar, 10 cm. **h**,**k**,**n**, Disease symptoms and lesion lengths of the wild-type, *osd2* (**h**), *osd3* (**k**) and *osd4* (**n**) plants at 14 dpi. Scale bar, 3 cm. **i**,**l**,**o**, The SA and SAG contents in the leaves of 14-day-old plants of wild-type, *osd2* (**i**), *osd3* (**l**) and *osd4* (**o**) plants. Data are mean ± s.d.; *n* = 20 (**b**,**h**,**k**,**n**) and *n* = 4 (**c**,**i**,**l**,**o**) biologically independent samples. Statistical analysis by two-sided Student’s *t*-test (**b**,**c**,**h**,**i**,**k**,**l**,**n**,**o**). All experiments were repeated at least twice with similar results. Source Data

To elucidate the PAL-SA pathway, we conducted a high-throughput forward genetic screen for SA-deficient mutants from rice ethyl methyl sulfonate (EMS)-mutant libraries. This process was achieved using an engineered non-pathogenic bacterial biosensor, *Acinetobacter* sp. ADP1-derived SA biosensor (ADPWH_*lux*), which generates bioluminescence proportionally in response to salicylates over a wide range of concentrations^36,37^. Plant crude extracts were incubated with the bacterial biosensor and detected using a multimode microplate reader. Mutants exhibiting significantly reduced luminescence intensity compared to the wild type were further analysed using high-performance liquid chromatography to assess the potential changes in SA levels. Two allelic mutants, designated as *O. sativa sa deficient 1* (*osd1-1*) and *osd1-2*, were identified from an EMS-mutagenized population of rice cultivar Xiushui 11 (XS11). The *osd1-1* and *osd1-2* mutants showed normal growth but exhibited a longer lesion than their parental plant XS11 after *Xanthomonas oryzae* pv. *oryzae* (*Xoo*) inoculation (Fig. 1a,b). The levels of free SA and SA-2-*O*-β-d-glucoside (SAG) were reduced to 5% and 1% of those in XS11, respectively, suggesting that *osd1-1* and *osd1-2* are SA-deficient mutants (Fig. 1c). We then backcrossed *osd1-1* with the WT and generated a BC_1_F_2_ population. Quantification of SA levels in 179 BC_1_F_2_ individuals revealed 135 lines with the wild-type phenotype, and 44 lines phenocopying *osd1-1*, supporting the idea that *osd1* is caused by a single nuclear gene mutation (*χ*^2^ = 0.017; *P* > 0.05 for the 3:1 hypothesis).

**Extended Data Fig. 1:**
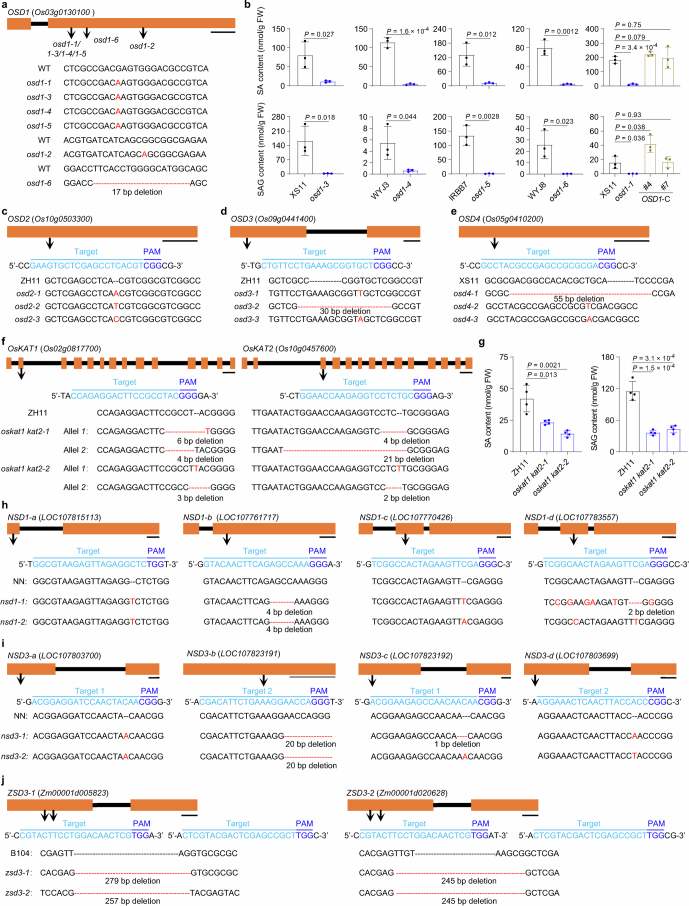
Characterization of the SA-deficient mutants of rice *osd1, osd2, osd3, osd4, oskat1 kat2*, tobacco *nnd1, nnd3*, and maize *zsd3.* **a**, Characterization of the allelic mutants of *osd1*. **b**, Quantification of SA and SAG contents in the leaves from adult plants of wild type (WT), *osd1-2*, *osd1-3*, *osd1-4*, *osd1-5*, and *OSD1*-C. FW, fresh weight. *OSD1*-C, the *osd1-1* mutant complemented by *OSD1*. **c**-**f**, Characterization of *osd2* (**c**), *osd3* (**d**), *osd4* (**e**) and *oskat1 kat2* double mutants (**f**) generated by CRISPR/Cas9 technology. **g**, Quantification of SA and SAG contents in the leaves from the adult plants of ZH11 and *oskat1 kat2* double mutant, Data are means ± s.d.; n = 3 (**b**) and n = 4 (**g**) biologically independent samples. Statistical analysis was performed using two-sided Student’s *t*-test. Exact *P* values are provided in the figure. All experiments were repeated at least twice with similar results. **h-j**, Characterization of *nsd1* (**h**), *nsd3* (**i**), and *zsd3* (**j**) mutants generated by CRISPR/Cas9 technology. The mutation nucleotides are shown in red font. The target site and the rotospacer-adjacent motif (PAM) are shown in light and dark blue font respectively. The orange rectangles indicate the gene coding region and the intermediate lines indicate introns. Scale bars, 200 bp. SA, salicylic acid; SAG, SA-2-*O*-β-D-glucoside. Source Data

To clone the *OSD1* gene, we mapped it to a physical interval of 1.038 Mb between the markers M4 and M5 by a map-based cloning approach (Fig. 1d). Then, we performed bulk population sequencing and identified the region on chromosome 3 from 1.62 to 1.64 Mb with single nucleotide polymorphism (ΔSNP) index = 0.86 (statistical significance under the null hypothesis: *P* < 0.01) (Fig. 1e). Within this latter interval, the annotated gene Os*03g0130100*, with a G672 base mutation to A base resulting in Glu180 mutation to Lys in *osd1-1*, was predicted to be the candidate gene, which encodes a putative cinnamoyl-CoA ligase, *Os*CNL1^38^. In addition, we identified five additional allelic *osd1* mutants (Fig. 1f and Extended Data Fig. 1a). All the allelic mutants exhibited SA-deficient phenotype, and the SA-deficiency of *osd1-1* was restored to the wild-type level by *OSD1* (Extended Data Fig. 1b). These data confirmed that *OSD1* (also known as Os*CNL1*) is the casual gene of the *osd1-1* mutant and, consistent with a recent report^29^, it is involved in SA biosynthesis.

Subsequently, we performed co-expression analysis with ATTED-II (http://atted.jp/; v.11.1)^39^ using *OSD1* as bait to explore further the unknown genes involved in SA biosynthesis. Among the top 50 ranked co-expressed genes from the microarray-based datasets (Supplementary Table 1), two PAL-like genes—Os*02g0627100* (also known as Os*PAL06*) and Os*04g0518400* (also known as Os*PAL07*)—were found, which aligns well with previous report that *Os*PAL06 is involved in SA biosynthesis in rice^40^. Since SA has long been hypothesized to be synthesized from BA^32^, co-expressed genes with potential functions in SA and BA metabolism were selected and knocked out using CRISPR–Cas9 gene editing (Extended Data Fig. 1c–e). Among them, the knockout mutants of Os*10g0503300* (putative BEBT gene), Os*09g0441400* (putative cytochrome P450 71A1 gene), and Os*05g0410200* (putative carboxylesterase gene) showed normal growth but were susceptible to *Xoo* (Fig. 1g,h,j,k,m,n). The lesion lengths in the leaves of the mutants were 30–60% longer than those of the wild type after *Xoo* inoculation (Fig. 1h,k,n). The SA and SAG levels in the mutants decreased to 0.36% and 0.50% of the wild-type levels, respectively (Fig. 1i,l,o). For simplicity, we named these recognized genes *OSD2* (Os*10g0503300*), *OSD3* (Os*09g0441400*) and *OSD4* (Os*05g0410200*). Together, our genetic data suggest that OSD1, OSD2, OSD3 and OSD4 are involved in SA biosynthesis.

## Biosynthesis of BA-CoA from *trans*-CA

Building on our genetic identification of OSD1 and previous findings that *Os*CNL and AIM1 are involved in SA biosynthesis in rice^23,25,29^, we sought to establish the potential biosynthetic pathway from *trans*-CA to SA in rice. We deduced that PAL-SA pathway probably comprises two key steps: (1) the synthesis of BA derivatives from *trans*-CA by β-oxidation; and (2) the synthesis of SA from BA derivatives by hydroxylation.

Previous studies indicated that BA-CoA is synthesized from *trans*-CA catalysed by CNL, producing cinnamoyl-CoA (CA-CoA), CHD, benzoylacetyl-CoA (BAc-CoA), followed by a further conversion to BA-CoA, catalysed by KAT^22,30^. To establish the β-oxidative pathway of BA-CoA biosynthesis in rice, in addition to CNL and AIM1, we searched the corresponding KAT enzyme. Using the Ph*KAT* gene to BLAST search rice genome enabled us to identify two rice KAT homologues, Os*02g0817700* (designated Os*KAT1*) and Os*10g0457600* (Os*KAT2*). The *oskat1 kat2* double mutants generated by CRISPR–Cas9 gene editing displayed significantly reduced SA content (Extended Data Fig. 1f,g). We then examined the transcriptional expression, subcellular localization, and biochemical properties of the recognized rice β-oxidative pathway enzymes OSD1, AIM1 and *Os*KAT1/KAT2.

**Fig. 2:**
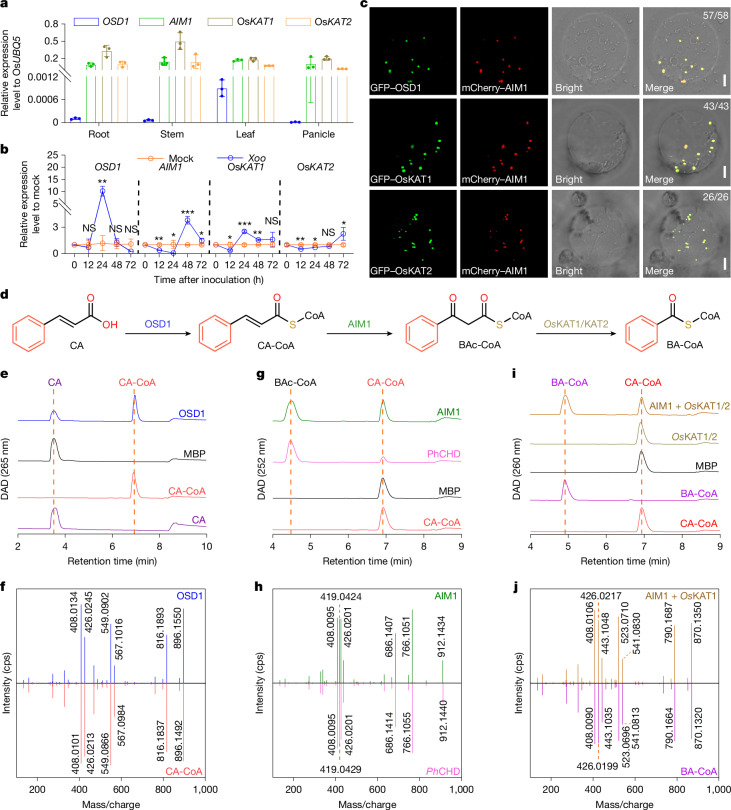
Biosynthesis of BA-CoA from *trans*-CA by the peroxisomal enzymes OSD1, AIM1 and *Os*KAT1/KAT2. **a**, Relative expression of *OSD1*, *AIM1*, Os*KAT1* and Os*KAT2* in the root, stem, leaf and panicle of 70-day-old ZH11 plants. **b**, Relative expression of *OSD1*, *AIM1*, Os*KAT1* and Os*KAT2* in the leaves of the ZH11 plants at 12 h, 24 h, 48 h and 72 h after *Xoo* inoculation. Gene expression in the *Xoo-*infected group was normalized to the mock group. Data are mean ± s.d.; *n* = 3 (**a**,**b**) biologically independent samples. Statistical analysis by two-sided Student’s *t*-test (**b**). *P < 0.05, **P < 0.01, ***P < 0.001; NS, not significant. Exact *P* values are presented in Supplementary Table 2. **c**, Subcellular localization of OSD1, *Os*KAT1 and *Os*KAT2 in rice protoplast. AIM1, a peroxisome-localized enzyme, was used as a peroxisomal marker. Scale bars, 5 μm. The fraction of protoplasts showing the localization pattern over total co-transformed protoplasts analysed is shown at the top right of each merged image. **d**, Biosynthetic pathway for BA-CoA from *trans*-CA in rice. **e**, Diode array detector detection (DAD) chromographs of the reaction from CA to CA-CoA catalysed by the CA-CoA ligase OSD1. **f**, MS/MS fragmentation pattern of the OSD1 enzymatic product and CA-CoA standard. **g**, DAD chromographs of the reaction from CA-CoA to BAc-CoA catalysed by the CA-CoA hydrogenase-dehydrogenase AIM1. **h**, MS/MS fragmentation pattern of the AIM1 enzymatic product and BAc-CoA, an enzymatic product of *Ph*CHD. **i**, DAD chromographs of the reaction from CA-CoA to BA-CoA catalysed by AIM1 and the 3-ketoacyl-CoA thiolases *Os*KAT1/KAT2. **j**, MS/MS fragmentation pattern of the enzymatic product of AIM1 coupled with OsKAT1 and BA-CoA standard. MS fragmentation patterns in **f**,**h**,**j** are characterized by liquid chromatography–tandem mass spectrometry (LC–MS/MS) with triple time of flight (TOF). All experiments were repeated at least twice with similar results. Source Data

First, we determined the tissue and organ-specific expression pattern of *OSD1*, *AIM1*, Os*KAT1* and Os*KAT2* in the root, leaf, stem and panicle of rice using quantitative PCR with reverse transcription (RT–qPCR). The results showed that these genes were expressed in all tested tissues, but *OSD1* exhibited the lowest expression levels, with approximately 0.1% the level of *AIM1* (Fig. 2a). The transcript levels of *OSD1*, *AIM1*, Os*KAT1* and Os*KAT2* were significantly induced up to 10.1-, 3.8-, 2.5- and 2.3-fold, respectively, after *Xoo* inoculation (Fig. 2b), implicating their possible functions in plant immunity. The GFP–OSD1, GFP–*Os*KAT1 and GFP–*Os*KAT2 fusion proteins, when co-expressed with the known peroxisomal protein mCherry–AIM1 (ref. ^23^) in the prepared rice protoplasts, showed overlapping fluorescence distribution patterns (Fig. 2c), indicating their co-localization in the peroxisome.

**Extended Data Fig. 2:**
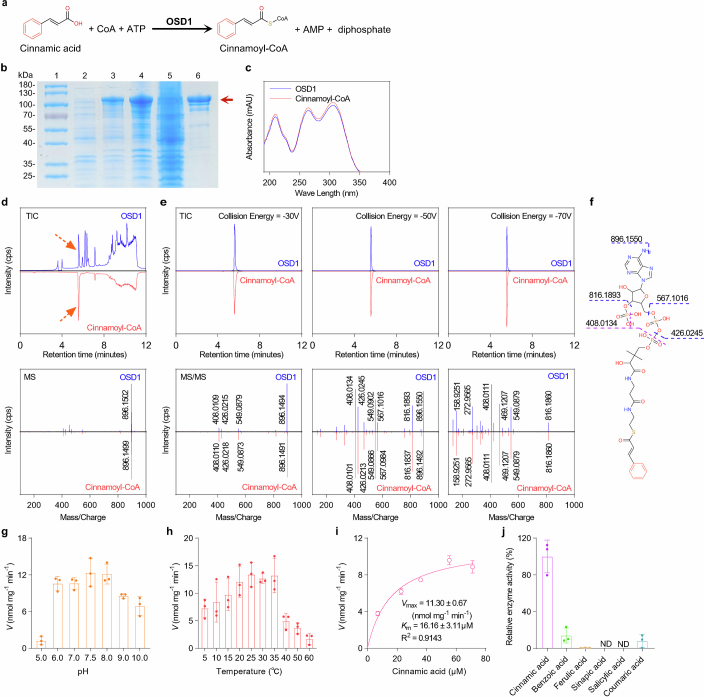
Biochemical assays of the recombinant OSD1 enzyme. **a**, Biochemical reaction catalyzed by the cinnamoyl-CoA ligase OSD1. **b**, Expression and protein purification of the recombinant MBP-OSD1 in *E. coli* BL21 (DE3). Lane 1, the molecular mass ladder; lanes 2 and 3, the crude extracts from *E. coli* with MBP-OSD1 untreated or treated with IPTG respectively; the soluble fractions (lane 4) and insoluble fractions (lane 5) from sonicated *E. coli* with MBP-OSD1 treated with IPTG; lanes 6, the purified recombinant MBP-OSD1 protein. The arrow points to the MBP-OSD1 protein. Proteins were visualized by Coomassie Blue R-250 staining. **c**, UV spectra of the OSD1 enzymatic product and CA-CoA standard. **d** and **e**, The LC total ion chromatogram and MS1 pattern (**d**), and specific ion chromatograms and MS-MS pattern (**e**) of the OSD1 enzymatic product and CA-CoA standard at varying collision energies. The obtained molecular ion [M-H]^−^ at m/z 896.1502 was an indicative peak for CA-CoA formation. **f**, The structure and proposed fragments of OSD1 products. **g** and **h**, Effects of pH value (**g**) and temperature (**h**) on enzyme activities of recombinant protein MBP-OSD1. The optimal temperature is around 20 °C, and the optimal pH value is 7.5. **i**, Kinetics of the recombinant MBP-OSD1 protein. The enzymatic activity obeys Michaelis-Menten equation. **j**, Substrate specificities assays of the recombinant MBP-OSD1 toward different substrates including *trans*-cinnamic acid, benzonic acid, ferulic acid, sinapic acid, salicylic acid and coumaric acid. Data are means ± s.d.; n = 3 (**g**, **h**, **i**, and **j**) independent samples. All experiments were repeated at least twice with similar results. Source Data

**Extended Data Fig. 3:**
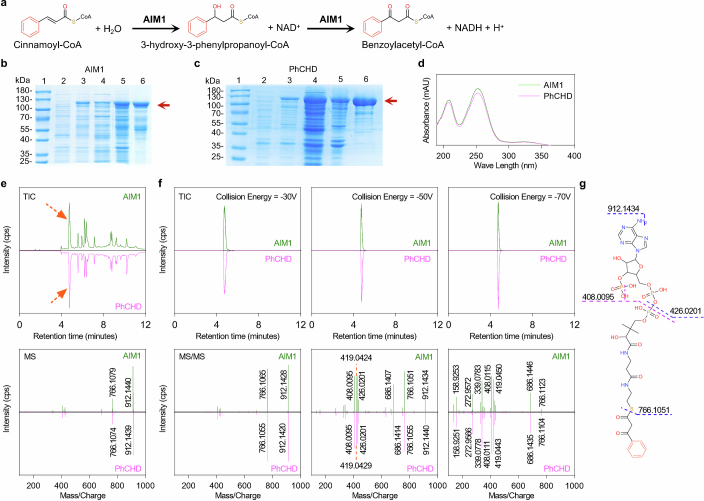
Biochemical assays of the recombinant AIM1 enzyme. **a**, Biochemical reactions catalyzed by the cinnamoyl-CoA hydrogenase-dehydrogenase AIM1. **b** and **c**, Protein expression purification of the recombinant protein MBP-AIM1 (**b**) and MBP-PhCHD (**c**) in *E. coli* BL21 (DE3). Lane 1, the molecular mass ladder; lanes 2 and 3, the crude extracts from *E. coli* with MBP-AIM1 (**b**) and MBP-PhCHD (**c**) without or with IPTG treatment; the insoluble fractions (lane 4) and soluble fractions (lane 5) from sonicated *E. coli* with MBP-AIM1 (**b**) and MBP-PhCHD (**c**) treated with IPTG; lanes 6, the purified recombinant MBP-AIM1 (**b**) and MBP-PhCHD (**c**) protein. The arrow points to the MBP-AIM1 (**b**) and MBP-PhCHD (**c**) proteins. Proteins were visualized by Coomassie Blue R-250 staining. PhCHD, a known CA-CoA hydrogenase-dehydrogenase form petunia (*Petunia hybrida*) as a positive control. **d**, UV spectra of the enzymatic product of MBP-AIM1 and BAc-CoA, a known product of MBP-PhCHD. **e** and **f**, The LC total ion chromatogram and MS1 pattern (**e**), and specific ion chromatograms and MS/MS patterns at multiple collision energies (**f**). The obtained molecular ion [M-H]^−^ at m/z 912.1440 was an indicative peak for BAc-CoA formation. **g**, The structure and proposed fragments of AIM1 enzymatic products. All experiments were repeated at least twice with similar results.

**Extended Data Fig. 4:**
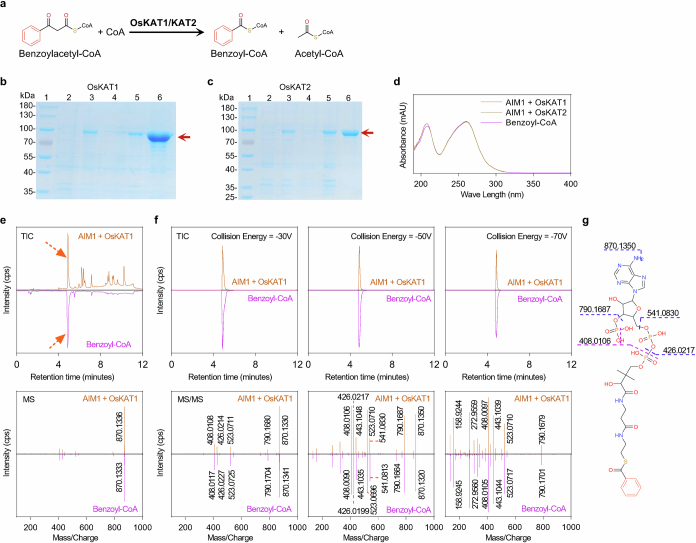
Biochemical assays of the recombinant OsKAT1/KAT2 enzyme coupled with AIM1. **a**, Biochemical reaction catalyzed by the 3-ketoacyl-CoA thiolases OsKAT1/KAT2. **b** and **c**, Protein expression and purification of the recombinant protein MBP-OsKAT1 (**b**) or MBP-OsKAT2 (**c**) in *E. coli* BL21 (DE3). Lane 1, the molecular mass ladder; lanes 2 and 3, the crude extracts from *E. coli* with MBP-OsKAT1 (**b**) or MBP-OsKAT2 (**c**) without or with IPTG treatment; the insoluble fractions (lane 4) and soluble fractions (lane 5) from sonicated *E. coli* with MBP-OsKAT1 (**b**) or MBP-OsKAT2 (**c**) treated with IPTG; lanes 6, the purified recombinant MBP-OsKAT1 (**b**) or MBP-OsKAT2 (**c**) protein. The arrow points to the MBP-OsKAT1 (**b**) or MBP-OsKAT2 (**c**) proteins. Proteins were visualized by Coomassie Blue R-250 staining. **d**, UV spectra of the MBP-AIM1 and MBP-OsKAT1/KAT2 coupling enzymatic product and BA-CoA standard. **e** and **f**, The LC total ion chromatogram and MS1 pattern (**e**), and specific ion chromatograms and MS-MS patterns (**f**) at multiple collision energies. The obtained molecular ion [M-H]^−^ at m/z 870.1336 was an indicative peak for BA-CoA formation. **g**, The structure and proposed fragments of AIM1 and OsKAT1 coupling enzymatic products. All experiments were repeated at least twice with similar results.

We then prepared recombinant OSD1, AIM1 and *Os*KAT1/KAT2 enzymes in *Escherichia coli* and conducted biochemical analyses to validate the proposed BA-CoA biosynthetic pathway (Fig. 2d and Extended Data Figs. 2a, 3a and 4a). With purified recombinant OSD1 protein (Extended Data Fig. 2b), the substrate *trans*-CA was efficiently converted to CA-CoA, as detected by liquid chromatography–ultraviolet array–mass spectrometry (LC–UV–MS) analysis (Fig. 2e,f). The identity of the enzymatic product was confirmed by comparing its retention time, UV spectrum, mass spectrum, and tandem mass spectrometry (MS/MS) fragmentation patterns with those of authentic CA-CoA standard (Fig. 2e,f and Extended Data Fig. 2c–f). These results confirm that OSD1 catalyses the formation of CA-CoA. Kinetics analysis of OSD1 revealed that the enzyme followed a Michaelis–Menten equation (Extended Data Fig. 2g–i). Substrate specificity analysis showed that OSD1 exhibited the highest activity towards *trans*-CA among the tested substrates, although weak (or no) activity was observed for BA, ferulic acid, coumaric acid, SA or sinapic acid (Extended Data Fig. 2j).

Although AIM1 in rice has been associated with the β-oxidation pathway in SA biosynthesis^14,23^, its enzyme activity remains to be characterized. We heterogeneously expressed and purified the recombinant AIM1 protein from *E. coli* (Extended Data Fig. 3b). The enzymatic products were detected and identified by LC–UV–MS. Since no authentic BAc-CoA standard was commercially available, we purified the recombinant protein of the previously characterized *Ph*CHD^25^ from *E. coli* (Extended Data Fig. 3c) and used it to produce a BAc-CoA standard. The enzymatic product of AIM1 showed the same retention time, UV and mass spectra, and MS/MS fragmentation pattern as the *Ph*CHD enzymatic product (Fig. 2g,h and Extended Data Fig. 3d–g), confirming the product of AIM1 as BAc-CoA. Thus, AIM1 functions as a CA-CoA hydratase-dehydrogenase for converting CA-CoA to BAc-CoA.

The recombinant *Os*KAT1/KAT2 proteins, heterogeneously expressed and purified from *E. coli* (Extended Data Fig. 4b,c), were then coupled with AIM1 to measure the enzymatic activity converting CA-CoA to BAc-CoA, and then to BA-CoA (Extended Data Figs. 3a and 4a). The enzymatic product of the coupling assay displayed the same retention time, UV spectrum, mass spectrum and MS/MS fragmentation pattern as an authentic BA-CoA standard (Fig. 2i,j and Extended Data Fig. 4d–g). Without coupling with AIM1, *Os*KAT1/KAT2 did not catalyse the conversion of CA-CoA to BA-CoA (Fig. 2i). Collectively, these results elucidate the β-oxidative pathway for BA-CoA biosynthesis in rice, sequentially catalysed by OSD1, AIM1 and *Os*KAT1/KAT2.

## Biosynthesis of SA from BA-CoA

It has been hypothesized that SA can be synthesized from BA derivatives by hydroxylation^32^. Since the knockout mutants of *OSD2*, *OSD3* and *OSD4* showed an SA-deficient phenotype (Fig. 1i,l,o), we examined their functions in the hydroxylation of BA derivatives to SA.

**Fig. 3:**
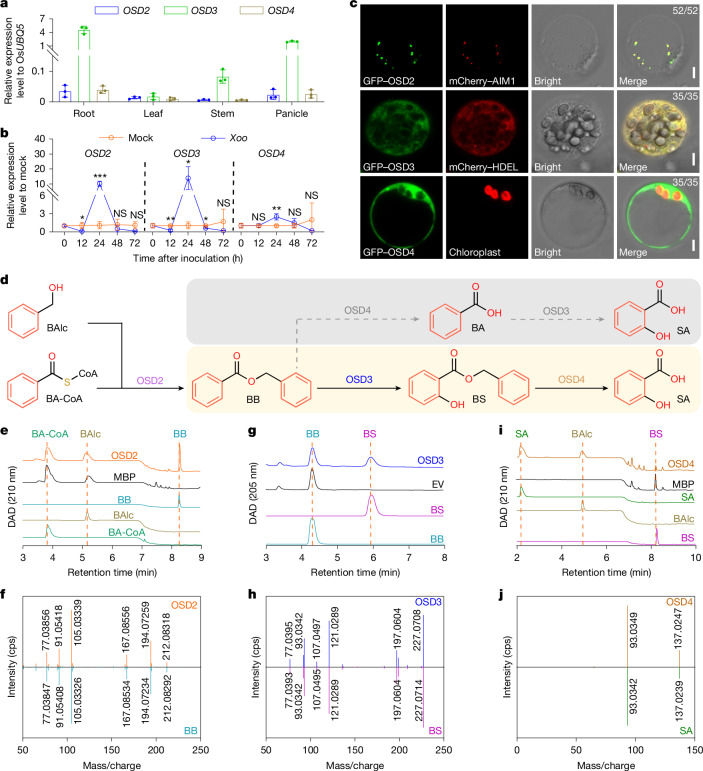
Biosynthesis of SA from BA-CoA by the peroxisomal OSD2, endoplasmic reticulum-resident OSD3 and cytoplasmic OSD4 enzymes. **a**, Expression of *OSD2*, *OSD3* and *OSD4* in the root, stem, leaf and panicle of 70-day-old ZH11 plants. **b**, Expression of *OSD2*, *OSD3* and *OSD4* in the leaves of ZH11 plants at 12 h, 24 h, 48 h and 72 h after *Xoo* inoculation. Gene expression in the *Xoo*-infected group was normalized to the mock group. Data are mean ± s.d.; *n* = 3 (**a**,**b**) biologically independent samples. Statistical analysis by two-sided Student’s *t*-tests (**b**). Exact *P* values are presented in Supplementary Table 2. **c**, Subcellular localization of OSD2, OSD3 and OSD4 in rice protoplast. mCherry–HDEL is an endoplasmic reticulum-localized marker. Scale bars, 5 μm. The fraction of protoplasts showing the localization pattern to total co-transformed protoplasts analysed is shown at the top right of each merged image. **d**, Two hypothetical routes for SA biosynthesis from BA-CoA in rice. **e**, DAD chromographs of the reaction from BA-CoA and BAlc to BB catalysed by the BA-CoA:benzyl alcohol benzoyltransferase OSD2. **f**, MS fragmentation (GC Orbitrap MS) pattern of the OSD2 enzymatic product and BB standard. **g**, DAD chromographs of the reaction from BB to BS catalysed by BB 2-hydroxylase OSD3. **h**, MS/MS fragmentation (LC–MS/MS, triple TOF) pattern of the OSD3 enzymatic product and BS standard. **i**, DAD chromographs of the reaction from BS to SA catalysed by the BS carboxylesterase OSD4. **j**, MS/MS fragmentation (LC–MS/MS) pattern of the OSD4 enzymatic product and SA standard. All experiments were repeated at least twice with similar results. Source Data

We analysed the expression patterns of *OSD2*, *OSD3* and *OSD4* genes in the root, leaf, stem and panicle, or under pathogen treatment, by RT–qPCR. Similar to *OSD1*, *AIM1* and Os*KAT1*/*KAT2*, the expression of *OSD2*, *OSD3* and *OSD4* was ubiquitous in rice (Fig. 3a). The expression levels of *OSD2*, *OSD3* and *OSD4* were significantly increased, showing up to 10.1-, 13.9- and 2.4-fold increases, respectively, after *Xoo* inoculation (Fig. 3b). Co-expression of GFP–OSD2 and mCherry–AIM1 in rice protoplasts showed co-localization within all the detected protoplasts, suggesting that OSD2, similar to the tobacco BEBT^27^, is localized in the peroxisome. Similarly, co-localization of GFP–OSD3 with the endoplasmic reticulum marker mCherry–HDEL^41^ and co-immunoblotting with binding protein (BiP)^42^ indicates its localization in the endoplasmic reticulum membrane (Fig. 3c and Extended Data Fig. 6a). The ubiquitously distributed signal of GFP–OSD4 suggests that OSD4 is localized in the cytosol (Fig. 3c).

**Extended Data Fig. 5:**
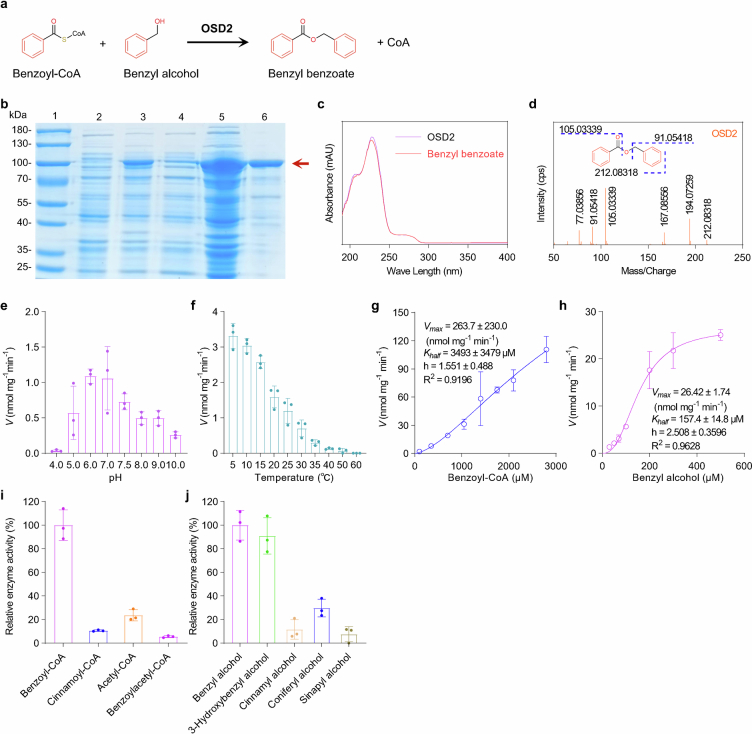
Biochemical assays of the recombinant OSD2 enzyme. **a**, Biochemical reaction catalyzed by the benzoyl-CoA:benzylalcohol benzoyltransferase OSD2. **b**, Protein expression and purification of the recombinant MBP-OSD2 in *E. coli* Rosetta 2 (DE3). Lane 1, the molecular mass ladder; lanes 2 and 3, the crude extracts from *E. coli* with MBP-OSD2 without or with IPTG treatment; the insoluble fractions (lane 4) and soluble fractions (lane 5) from sonicated *E. coli* with MBP-OSD2 treated with IPTG; lanes 6, the purified recombinant MBP-OSD2 protein. The arrow points to the MBP-OSD2 proteins. Proteins were visualized by Coomassie Blue R-250 staining. **c**, UV spectra of the OSD2 enzymatic product and BB standard. **d**, MS pattern of the OSD2 enzymatic products analyzed by GC Orbitrap MS. The structure and proposed fragments of OSD2 products enzymatic were shown. **e** and **f**, Effects of pH value (**e**) and temperature (**f**) on enzyme activities of MBP-OSD2. The optimal temperature is around 5–10 °C, and the optimal pH value is 6.0. **g** and **h**, Kinetics of the recombinant MBP-OSD2 protein on BA-CoA (**g**) or BAlc (**h**). The enzymatic kinetics obeys allosteric sigmoidal equation. **i** and **j**, Substrate specificities assays of the recombinant MBP-OSD2 toward different CoA substrates (**i**) including BA-CoA, CA-CoA, Acetyl-CoA, and BAc-CoA, and different alcohol (**j**) including benzyl alcohol, 3-hydroxybenzyl alcohol, cinnamic alcohol, coniferyl alcohol, and sinapyl alcohol. Data are means ± s.d.; n = 3 (**e**, **f**, **g**, **h**, **i**, and **j**) independent samples. CA-CoA, cinnamoyl-CoA; BAc-CoA, benzoylacetyl-CoA; BA-CoA, benzoyl-CoA; BAlc, benzyl alcohol; BB, benzyl benzoate. Source Data

OSD2 is annotated as a putative BEBT (Fig. 3d, Supplementary Table 1 and Extended Data Fig. 5a). To validate its function, we heterogeneously expressed and purified the recombinant OSD2 protein from *E. coli* (Extended Data Fig. 5b) and conducted an enzymatic assay by incubating it with BA-CoA and benzyl alcohol (BAlc). The products were detected by LC–UV–MS and gas chromatography–mass spectrometry (GC–MS). The retention time, UV spectrum and mass spectrum of the OSD2 enzymatic product matched those of the authentic benzyl benzoate (BB) standard (Fig. 3e,f and Extended Data Fig. 5c,d). This product was not formed in the assays incubating purified MBP protein from *E. coli* containing the empty vector (Fig. 3e). These results indicate that OSD2 functions as a BEBT that catalyses the formation of BB from BA-CoA and BAlc.

Kinetic analysis of OSD2 using BA-CoA or BAlc at different concentrations indicated that, unlike other BEBT enzymes^43–45^, OSD2 follows allosteric sigmoidal enzyme kinetics rather than Michaelis–Menten behaviour (Extended Data Fig. 5e–h). This kinetic behaviour suggests positive cooperativity in enzyme activity towards BA-CoA or BAlc, implying that the OSD2 activity may be regulated by the cellular concentration of BA-CoA and BAlc. Substrate specificity assays showed that OSD2 predominantly uses BA-CoA as the thioester donor and prefers BAlc over other tested phenol alcohols as the acceptor (Extended Data Fig. 5i,j).

**Extended Data Fig. 6:**
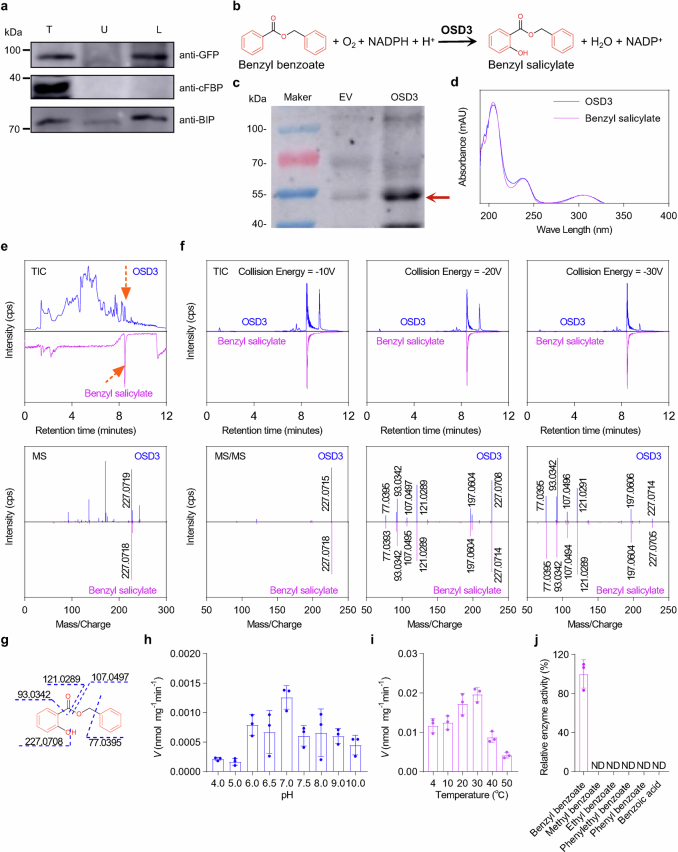
Subcellular localization of OSD3 and biochemical assays of the recombinant OSD3 enzyme. **a**, Immunoblot analysis of endoplasmic reticulum (ER) fractions from GFP-OSD3 expressing *N. benthamiana* by probing with antibodies against GFP, cFBP (CFBPase, cytoplasmic marker) and BIP (BIP1/2, ER marker). T, total protein; U, upper phase (plasma membrane); L, lower phase (internal membranes). **b**, Biochemical reaction catalyzed by the benzyl benzoate 2-hydroxylase OSD3. **c**, Protein extraction of the recombinant protein Flag-OSD3 in yeast strain WAT11. Lane 1, the molecular mass ladder; lanes 2 and 3, the microsomal extracts from the yeast with empty vector (EV) or Flag-OSD3, respectively. The recombinant protein Flag-OSD3 in microsomal extracts were visualized by probing with antibodies against Flag tag. The arrow points to the Flag-OSD3 proteins. For gel source data of (**a**) and (**c**), see Supplementary Fig. 1. **d**, UV spectra of the OSD3 enzymatic product and BS standard. **e** and **f**, The LC total ion chromatogram and MS1 pattern (**e**), and specific ion chromatograms and MS-MS pattern (**f**) of the OSD3 enzymatic product and BB standard at multiple collision energies. The obtained molecular ion [M-H]^−^ at m/z 227.0719 was an indicative peak for BB formation. **g**, The structure and proposed fragments of OSD3 products. **h** and **i**, Effects of pH value (**h**) and temperature (**i**) on enzyme activities of the recombinant protein MBP-OSD3. The optimal temperature is around 30 °C, and the optimal pH value is 7.0. **j**, Substrate specificities assays of the recombinant MBP-OSD3 toward different substrates including BB, methyl benzoate, ethyl benzoate, phenylethyl benzoate, phenyl benzoate, and benzoic acid. The extract from empty vector (EV) yeast was the control. Data are means ± s.d.; n = 3 (**h**, **i**, and **j**) independent samples. BB, benzyl benzoate; BS, benzyl salicylate. Source Data

**Extended Data Fig. 7:**
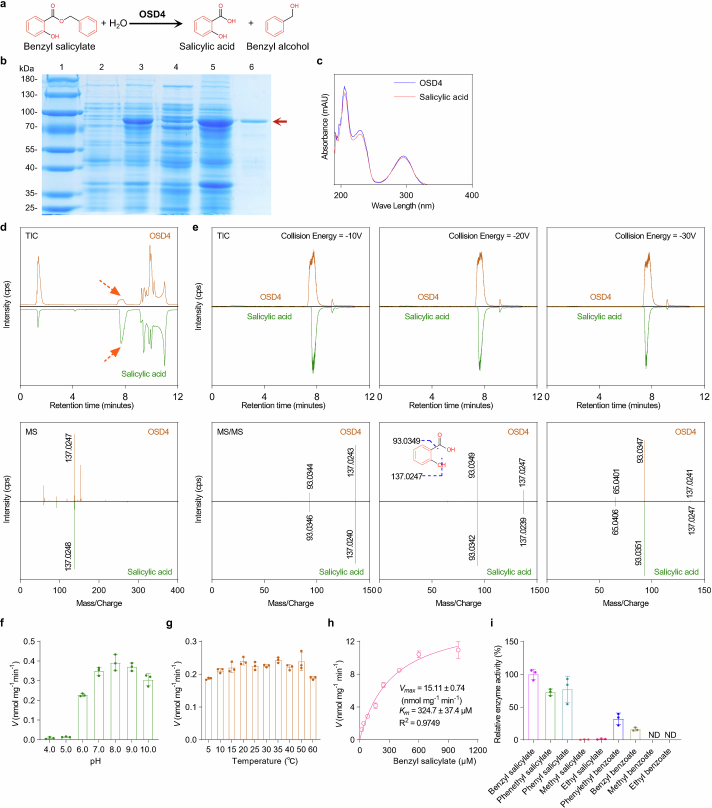
Biochemical assays of the recombinant OSD4 enzyme. **a**, Biochemical reaction catalyzed by the benzyl salicylate carboxylesterase OSD4. **b**, Protein expression and purification of the recombinant protein MBP-OSD4 in *E. coli* BL21 (DE3). Lane 1, the molecular mass ladder; Lanes 2 and 3, the crude extracts from *E. coli* with MBP-OSD4 without or with IPTG treatment; the insoluble fractions (Lane 4) and soluble fractions (lane 5) from sonicated *E. coli* with MBP-OSD4 treated with IPTG; lanes 6, the purified MBP-OSD4 protein. The arrow points to the MBP-OSD4 protein. Proteins were visualized by Coomassie Blue R-250 staining. **c**, UV spectra of the OSD4 enzymatic product and SA standard. **d** and **e**, The LC total ion chromatogram and MS1 pattern (**d**), and specific ion chromatograms and MS/MS patterns (**e**) at multiple collision energies. The obtained molecular ion [M-H]^−^ at m/z 137.0247 was an indicative peak for SA formation. The structure and proposed fragments of OSD4 enzymatic products were shown in (**e**). **f** and **g**, Effects of pH value (**f**) and temperature (**g**) on enzyme activities of MBP-OSD4. The optimal temperature is around 20–50 °C and the optimal pH value is 8.0. **h**, Kinetics of MBP-OSD4 protein on BS. The enzymatic activity obeys the Michaelis-Menten equation. **i**, The enzyme substrate specificities assays of recombinant MBP-OSD4 toward different substrates including benzyl salicylate, phenethyl salicylate, phenyl salicylate, methyl salicylate, ethyl salicylate, phenylethyl benzoate, benzyl benzoate, methyl benzoate, ethyl benzoate. Data are means ± s.d.; n = 3 (**f**, **g**, **h**, and **i**) independent samples. BS, benzyl salicylate; BAlc, benzyl alcohol; SA, salicylic acid. Source Data

OSD3 and OSD4 were annotated as a putative cytochrome P450 enzyme and a carboxylesterase, respectively (Supplementary Table 1). On the basis of their annotated functions, we hypothesized that BB could be transformed through two alternative routes for SA biosynthesis (Fig. 3d). First, it may be catalysed by OSD3 to produce benzyl salicylate (BS) (Extended Data Fig. 6b), which is then hydrolysed by OSD4 to produce SA (Extended Data Fig. 7a). Alternatively, it may be catalysed by OSD4 to produce BA, which is then hydroxylated by OSD3 to produce SA. To test this hypothesis, we performed enzyme assays of OSD3 and OSD4.

We expressed the recombinant OSD3 enzyme in yeast (*Saccharomyces cerevisiae*) and confirmed its expression by western blot analysis (Extended Data Fig. 6c). The microsomal fraction was prepared and used in an enzyme assay with BB as the substrate. The enzyme product was detected by LC–UV–MS and verified by comparing its retention time, UV spectrum, mass spectrum and MS/MS patterns with those of the authentic BS standard (Fig. 3g,h and Extended Data Fig. 6d–g). The data indicated that OSD3 acts as a benzyl benzoate 2-hydroxylase (BB2H) to catalyse the hydroxylation of BB, yielding BS. The pH and temperature conditions were optimized, and the substrate specificity assays were performed using other benzoate conjugates, including phenylethyl benzoate, phenyl benzoate, ethyl benzoate, methyl benzoate and BA. The results showed that OSD3 did not exhibit detectable activity towards BA or other tested substrates (Extended Data Fig. 6h–j), indicating its strict substrate specificity.

The recombinant OSD4 was heterogeneously expressed and purified from *E. coli* (Extended Data Fig. 7b). BS was tested in the OSD4 enzymatic assay. The reaction product showed similar retention time, UV spectrum, mass spectrum and MS/MS fragmentation pattern to authentic SA standard (Fig. 3i,j and Extended Data Fig. 7c–e), indicating that OSD4 acts as a benzyl salicylate hydrolase (BSH) to hydrolyse BS to SA. Further kinetic analysis revealed that OSD4 follows the typical Michaelis–Menten curve (Extended Data Fig. 7f–h). The enzyme exhibited limited substrate promiscuity. In addition to BS, it also hydrolysed phenylethyl salicylate, phenyl salicylate and BB. However, the activity towards BB was only about 20% of that towards BS (Extended Data Fig. 7i). These data suggest that SA is synthesized from BA-CoA via the following route: BA-CoA→BB→BS→SA (Fig. 3d), with each step sequentially catalysed by OSD2, OSD3 and OSD4.

## PAL-SA pathway in rice defence response

**Extended Data Fig. 8:**
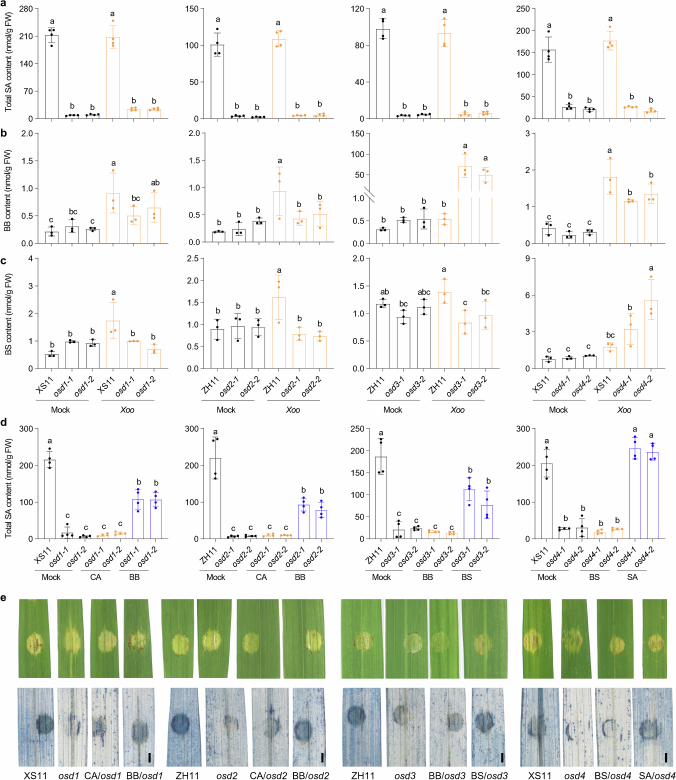
PAL-SA pathway is the primary SA biosynthetic route in rice disease response. **a**-**c**, The total SA (SA + SAG) (**a**), BB (**b**), and BS (**c**) contents in the leaves of the adult plants of WT (XS11 or ZH11) and *osd1*, *osd2*, *osd3*, and *osd4* mutant plants at 48 hrs after *Xoo* inoculation; **d**, The total SA (SA + SAG) contents in the leaves of adult plants of WT (XS11 or ZH11) and *osd1*, *osd2*, *osd3*, and *osd4* mutant treated with 200-μM CA, BB, BS or SA for 6 days; Data are means ± s.d.; n = 4 (**a** and **d**) and n = 3 (**b** and **c**) biologically independent samples. Statistical analysis was performed using one-way ANOVA of the LSD test, and the different lowercase letters represented the significant differences at the level of 0.05. **e**, Top images: Disease symptoms in the leaves of the WT (ZH11 and XS11), *OSD*-associated mutants, and the substrate- and product-fed mutants at 4 days post inoculation (dpi) by syringe-infiltrated *Xoo*. The brown spots indicate the cell death induced by hypersensitive response (HR), and the water-stained areas indicate susceptibility reaction. Bottom images: trypan blue staining of the leaves of the WT (ZH11 and XS11), *OSD*-associated mutants, and the substrate- and product-fed mutants at 3 dpi by syringe-infiltrated *Xoo*. The trypan blue-staining intensity of the infected leaves was used to visualize cell death levels in the leaves. Photos were captured by a stereo microscope. Scan bars, 2 mm. All experiments were repeated at least twice with similar results. SA, salicylic acid, SAG, SA-2-*O*-β-D-glucoside, BB, benzyl benzoate, BS, benzyl salicylate, CA, cinnamic acid. Source Data

To examine the role of PAL-SA pathway in SA biosynthesis during rice defence, we quantified SA contents in the wild type and *osd1–osd4* after pathogen *Xoo* infection. In agreement with a previous study^33^, SA levels in wild-type rice were high and not significantly induced by pathogens (Extended Data Fig. 8a). Moreover, regardless of pathogen infection, the deficiency of SA in the *osd* mutants remained essentially the same (Extended Data Fig. 8a). These data suggest that the PAL-SA pathway is the primary route for SA biosynthesis in rice.

Furthermore, we quantified the two main biosynthetic intermediates, BB and BS. The amounts of BB and BS were generally low with no significant difference between the *osd* mutants and the wild-type plant in the absence of pathogen treatment. Upon pathogen infection, the amount of BB slightly increased in the wild type, decreased in *osd1* and *osd2*, but increased approximately 100-fold in *osd3* (Extended Data Fig. 8b); similarly, the amount of BS slightly decreased in *osd1*, *osd2* and *osd3*, but was substantially increased in *osd4* (Extended Data Fig. 8c). We then conducted foliar spraying of CA, BB, BS or SA on wild-type, *osd1*, *osd2*, *osd3* and *osd4* plants, and assessed their abilities to rescue the low-SA and *Xoo-*susceptible phenotypes of the mutants (Extended Data Fig. 8d,e). The results demonstrated that feeding BB, but not CA, rescued *osd1* and *osd2*, confirming that OSD1 and OSD2 are essential for BB biosynthesis. Feeding BS, but not BB, rescued *osd3*, indicating that OSD3 is required for BS production from BB. Finally, feeding SA, but not BS, rescued *osd4*, verifying that OSD4 is essential for SA production from BS. These results collectively confirm that BB and BS are natural intermediates in the PAL pathway for primary SA biosynthesis in rice.

## Evolution of the PAL-SA pathway

**Fig. 4:**
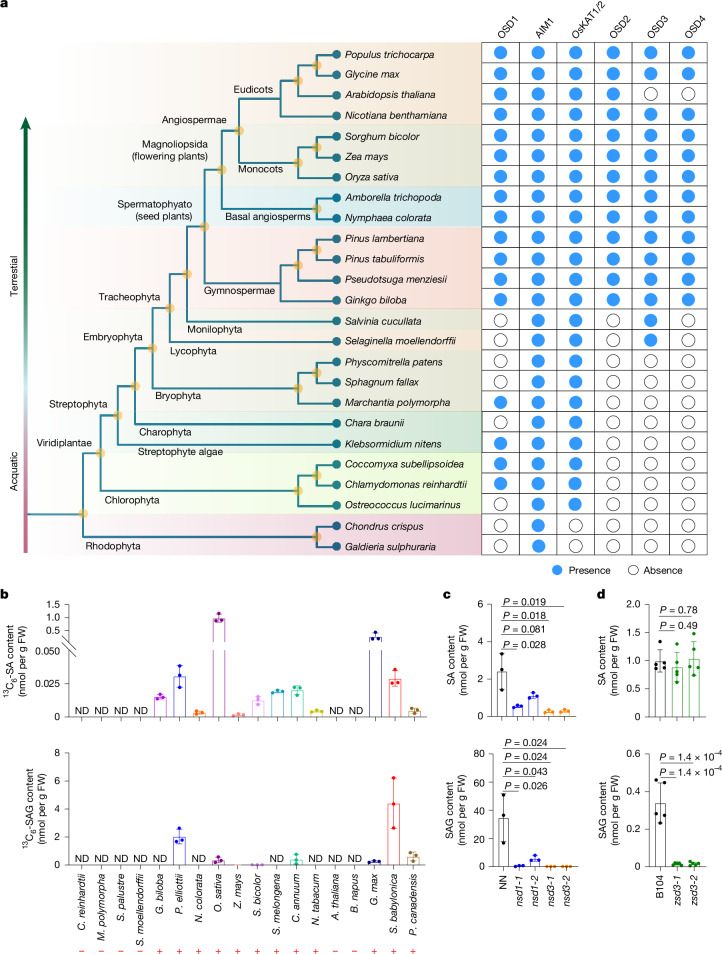
Evolutionary analysis of the PAL-SA pathway in plants. **a**, Evolutionary distribution of closely related homologues of the key components of the PAL-derived SA biosynthetic pathway, including OSD1, AIM1, *Os*KAT1/KAT2, OSD2, OSD3 and OSD4 in 25 representative plant species. **b**, Isotope tracer experiment using ^13^C_6_-Phe feeding in evolutionarily representative species. ^13^C_6_-SA and ^13^C_6_-SAG contents were quantified in plants fed with 200 μM ^13^C_6_-Phe for 72 h. Plus and minus represent detectable and indetectable ^13^C_6_ -labelled SA or SAG, respectively. ND, not detected. **c**, SA and SAG contents in mature leaves of wild-type tobacco NN, *nsd1* and *nsd3* quadruple mutants 8 h after inoculation with *Pseudomonas syringae* pv. tomato DC3000. **d**, SA and SAG contents in seedling leaves of wild-type B104 and *zsd3* double mutant maize. Data are mean ± s.d.; *n* = 3 (**b**,**c**), *n* = 5 (**d**) biologically independent samples. Statistical analysis by two-sided Student’s *t*-test. All experiments were repeated at least twice with similar results. Source Data

**Extended Data Fig. 9:**
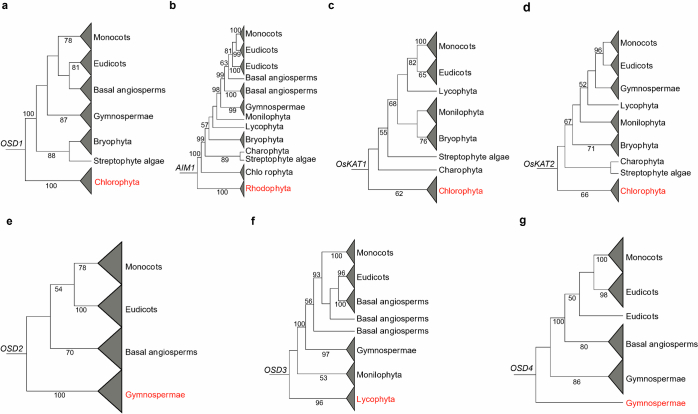
Simplified gene trees of the key components in PAL-SA pathway represented by taxa. The optimal models for maximum likelihood tree inferred by RAxML were JTT for *OSD1*, *OSD2*, *OSD3* and *OSD4*, and LGF for *AIM1* and *OsKAT1*/*KAT2*, respectively. The bootstraps above 50 were showed on branches. More than one representative taxa in gene trees may suggest potential events, such as gene duplication, loss, or incomplete lineage sorting. The triangle size represents the number of genes in each taxonomic group, and the most ancient taxon in which PAL-derived SA-biosynthetic key components originated was marked in red.

To understand the evolution of the PAL-SA pathway in plants, we performed comparative genomic analyses cross diverse plant species (Fig. 4a and Extended Data Fig. 9). Closely related homologues of *OSD1*, *AIM1*, Os*KAT1*/*KAT2*, *OSD2*, *OSD3* and *OSD4* were identified in 25 representative species spanning 11 major taxa, including Rhodophyta, Chlorophyta, Streptophyte algae, Charophyta, Bryophyta, Lycophyta, Monilophyta, Gymnospermae, basal angiosperms, Monocots and Eudicots (Fig. 4a and Extended Data Fig. 9). The homologues of these key components in the PAL-SA pathway were largely absent in Rhodophytes, except for AIM1. However, homologues of OSD1 and *Os*KAT1/KAT2 emerged in Chlorophyta, suggesting that the OSD1–AIM1–KAT1/KAT2-mediated β-oxidation pathway, which synthesizes benzoate derivatives, evolved and originated in green algae. Notably, OSD1 was lost in some early land plants after the divergence of terrestrial species. The cytochrome P450 enzyme OSD3 first appeared in the basal vascular plant *Selaginella* and is conserved in most tracheophyte plants. By contrast, OSD2 and OSD4 homologues emerged later with the appearance of gymnosperms and are largely conserved within seed plants. These findings suggest that the PAL-SA pathway evolved in a stepwise manner during plant evolution, with the complete functional pathway emerging prior to the divergence of gymnosperms. This pathway has been conserved across most seed plants, except for specific losses of OSD3 and OSD4 in *A. thaliana* (Fig. 4a).

To further confirm the presence of the PAL-SA pathway in plants, we conducted an isotope tracer experiment by feeding ^13^C-labelled phenylalanine (^13^C_6_-Phe) to evolutionarily representative species belonging to the unicellular green algae, primitive land plant bryophytes, basal vascular plants and several seed plants (Fig. 4b). The results revealed that ^13^C_6_-SA and/or ^13^C_6_-SAG were not detected in green algae, bryophytes and lycophytes, but were detected in most tested seed plants, except for *A. thaliana* and *Brassica napus*. The presence of the PAL-SA pathway in monocots and dicots was further validated in tobacco and maize using CRISPR–Cas9-derived mutants of tobacco *nsd1* and *nsd3* and maize *zsd3* (Fig. 4c,d and Extended Data Fig. 1h–j).

## Activation of the PAL-SA pathway in rice

**Fig. 5:**
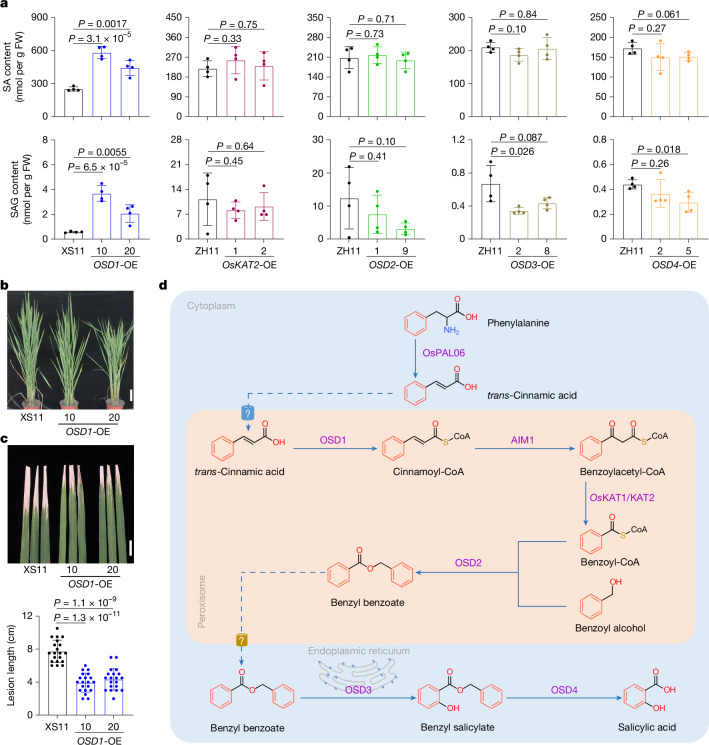
Activation of the PAL-SA pathway increases disease resistance in rice. **a**, SA and SAG contents in the seedlings of 14-day-old wild-type plants (XS11 or ZH11) and plants of the overexpression lines *OSD1*-OE, Os*KAT2*-OE, *OSD2*-OE, *OSD3*-OE and *OSD4*-OE. **b**, Phenotype of XS11 and pUbi-*OSD1*-pMDC32/*osd1-1* plants at the heading stage. Scale bar, 10 cm. **c**, Disease symptoms and lesion lengths in the leaves of wild-type (XS11) and *OSD1* overexpression lines at 14 dpi. Scale bar, 3 cm. Field-grown XS11 and pUbi-*OSD1*-pMDC32/*osd1-*1 plants were inoculated with *Xoo* at the tillering stage. Data are mean ± s.d.; *n* = 4 (**a**) and *n* = 20 (**c**) biologically independent samples. Statistical analysis by two-sided Student’s *t*-test (**a**,**c**). All experiments were repeated at least twice with similar results. **d**, Illustration of the PAL-SA pathway in rice. Solid arrows represent biochemical steps and their corresponding enzymes, and dashed arrows represent the compound translocation direction. OsPAL06, phenylalanine ammonia lyase; OSD1, cinnamoyl-CoA ligase; AIM1, cinnamoyl-CoA hydrogenase-dehydrogenase; *Os*KAT1/KAT2, 3-ketoacyl-CoA thiolase; OSD2, BA-CoA:benzyl alcohol benzoyltransferase; OSD3, benzyl benzoate 2-hydroxylase; OSD4, benzyl salicylate hydrolase. Source Data

**Extended Data Fig. 10:**
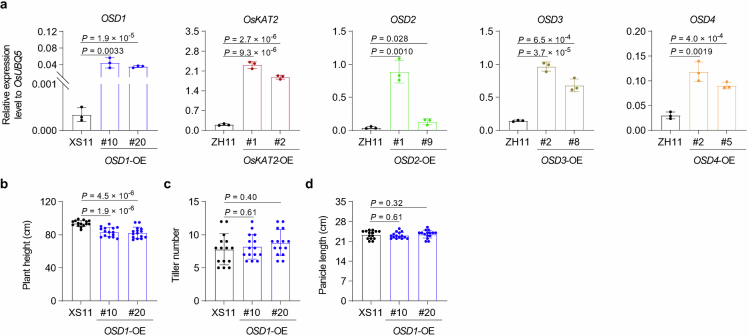
Characterization of the overexpression plants of *OSD1*, *OsKAT2*, *OSD2*, *OSD3*, and *OSD4* and the agronomic traits of the *OSD1* overexpression lines. **a**, The relative expression levels of *OSD1*, *OsKAT2*, *OSD2*, *OSD3*, *OSD4* in the overexpression rice *OSD1*-OE, *OsKAT2*-OE, *OSD2*-OE, *OSD3*-OE, and *OSD4*-OE at the tiller stage in the field. The expression level of each gene was normalized to the internal *OsUBQ5* gene. **b**-**d**, Plant height (**b**), tiller number (**c**), and panicle length (**d**) of the XS11 and *OSD1*-OE plants. Data are means ± s.d.; n = 3 (**a**) and n = 15 (**b**, **c**, and **d**) biologically independent samples. Statistical analysis was performed using two-sided Student’s *t*-tests. Exact *P* values are provided in the figure. All experiments were repeated at least twice with similar results. Source Data

To evaluate the role of rice PAL-SA pathway genes in plant immunity, we generated *OSD1*, Os*KAT2*, *OSD2*, *OSD3* and *OSD4* overexpression (OE) lines. RT–qPCR analyses confirmed that the expression levels of the transgenes were significantly increased in the respective lines (Extended Data Fig. 10a). However, a significant increase in SA levels (up to 230% compared with XS11) was observed only in the ubiquitin_pro_-*OSD1* overexpression lines (Fig. 5a). These findings suggest that *OSD1* might be one of the limiting factors in the PAL-SA pathway. Phenotypic analysis of *OSD1*-overexpression (OE) lines revealed that plant height was slightly reduced, whereas the tiller number and panicle length showed no significant differences compared with XS11 (Fig. 5b and Extended Data Fig. 10b–d). Notably, consistent with the elevated SA levels, the pathogen resistance of the *OSD1*-OE lines to *Xoo* infection was significantly increased compared with XS11 (Fig. 5c). These results demonstrate that activation of the PAL-SA pathway is an effective strategy to modulate crop pathogen resistance.

## Discussion

Growing evidence indicates that the PAL-based SA biosynthetic pathway is widespread in plants^6,7,11,14–24^. Previous studies suggest an association of the PAL-β-oxidation pathway with SA biosynthesis^14,23–30^. Nevertheless, the key enzymes responsible for converting BA derivatives to SA and the complete biosynthetic pathway originating from PAL-mediated phenylpropanoid metabolism remained unresolved. In this study, we identified four key enzymes and establish the complete phenylalanine-derived biosynthetic pathway of the plant stress hormone SA.

Through forward genetic screening for SA-deficient mutants, we obtained multiple allelic *osd1* mutants that were deficient in the CA-CoA ligase gene (Fig. 1f and Extended Data Fig. 1a). These *osd1* mutants exhibited a substantial reduction in SA levels before and after pathogen treatment (Fig. 1c and Extended Data Fig. 8a). Building on this foundation, we delineated the complete β-oxidation biosynthetic pathway for SA in rice peroxisome, which is composed of OSD1, AIM1 and *Os*KAT1/KAT2 (Figs. 1 and 2). Furthermore, we demonstrated that OSD2, a homologue of tobacco BEBT^27,30,31^, catalyses the conversion of BA-CoA to BB in the peroxisome, a reaction that is essential for SA biosynthesis in rice (Fig. 3e,f and Extended Data Fig. 5).

For decades, BA2H has been postulated to catalyse the conversion of BA to SA^32^. However, the precise identity of BA2H remains unknown, leaving a critical step in the SA biosynthetic pathway unresolved. Our findings challenge this longstanding hypothesis by demonstrating that the critical hydroxylation step occurs not at BA, but at BB. This reaction is catalysed by OSD3, an endoplasmic reticulum membrane-localized cytochrome P450 enzyme. OSD3 has a deduced molecular weight of 58.1 kDa (Extended Data Fig. 6c), distinctly different from the previously hypothesized 160-kDa soluble BA2H enzyme^32^. Enzyme assay using microsomal preparations revealed that OSD3 specifically hydroxylates BB to form BS, thereby functioning as a BB2H (Fig. 3g,h and Extended Data Fig. 6). These results underscore the involvement of multiple subcellular compartments in SA biosynthesis. BB is synthesized in the peroxisomes and probably translocated to the cytosol, where it is utilized by the endoplasmic reticulum membrane-bound BB2H enzyme OSD3 to produce BS. While previous studies suggested that BA-CoA may be exported from peroxisome to the cytosol for BB biosynthesis^25^. Our data and previous data showing that OSD2 and tobacco HSR201 reside in the peroxisome support the hypothesis that BB is produced in the peroxisome^27^. We speculate that, owing to its hydrophobic nature, BB may be more stable and more readily transportable across the peroxisome membrane than BA-CoA. The exact molecular mechanism governing the translocation of BB from the peroxisome to the cytosol remains unknown. In the cytosol, the BSH enzyme OSD4 hydrolyses BS to yield SA and BAlc (Fig. 3c,d,i,j). By unravelling the critical hydroxylation step of BB and the final hydrolysis step, we have now established the full PAL-SA pathway, tracing the process from CA to SA.

Previous evolutionary analyses of AIM1 indicated an ancient origin for the PAL-SA pathway^35^. Our study confirmed that the key enzymes constituting β-oxidation pathway for BA-CoA production, OSD1, AIM1 and *Os*KAT1/KAT2, evolved early, appearing as far back as chlorophyte green algae (Fig. 4a). By contrast, the emergence of OSD2, OSD3 and OSD4 occurred more recently. OSD3 probably evolved with basal vascular plants, whereas OSD2 and OSD4 emerged prior to the divergence of gymnosperms (Fig. 4a). These findings suggest that a fully functional PAL-SA pathway was not assembled until the emergence of gymnosperms. Our isotope trace experiments further support this conclusion, as ^13^C-labelled SA and derivatives were detected only in the fed seed plants (Fig. 4b). This stepwise-assembled pathway is largely conserved across seed plants with the notable exception of *A. thaliana*, in which OSD3 and OSD4 homologues are likely to have been lost (Fig. 4a). Consistent with a previous report in *A. thaliana*^46^, feeding ^13^C_6_-Phe failed to incorporate ^13^C into SA or SAG in both *A. thaliana* and *B. napus*, suggesting that SA biosynthesis in *Brassicaceae* may be lineage-specific. Additionally, the knockout mutants of the homologues of *OSD1* and *OSD3* in tobacco or *OSD3* in maize exhibited significantly reduced SA accumulation (Fig. 4c,d). These findings corroborate the idea that the PAL-SA pathway is conserved among seed plants.

Rice uniquely accumulates high levels of free SA. Consistent with previous reports^33^, our results show that SA levels do not significantly increase following pathogen infection (Extended Data Fig. 8a). This suggests that the PAL-SA pathway functions to maintain a constitutively high level of SA in rice independent of pathogen challenge. Given that SA is biologically active and potentially cytotoxic, the ability of rice cells to tolerate such high levels may point to mechanisms of spatial regulation, such as sequestration of SA within specific cellular compartments, including specialized organelles or biomolecular condensates. However, this hypothesis remains to be experimentally validated. In addition, the detection of low levels of the biosynthetic intermediates BB and BS, contrasted with the high concentration of SA in wild-type rice, implies the potential existence of metabolic channelling between key PAL-SA biosynthetic enzymes, such as OSD3 and OSD4. Such channelling could enable rapid and efficient conversion of intermediates to SA. This notion is further supported by our observation that overexpression of individual enzymes (OSD2, OSD3 and OSD4) did not significantly enhance SA accumulation (Fig. 5a and Extended Data Fig. 10), suggesting that their activities may rely on coordinated or compartmentalized interactions rather than on increased expression alone. Despite this, we found that overexpressing *OSD1* led to consistently elevated SA levels and enhanced pathogen resistance (Fig. 5a,c and Extended Data Fig. 10). This indicates that activating the PAL-SA pathway at the entry point can effectively boost SA-mediated immunity. On the basis of these insights, future strategies, such as engineering the elite allele of *OSD1* or driving its expression with stronger, pathogen-inducible promoters, could be used to enhance SA production and immunity in rice, while minimizing potential yield penalties.

In summary, we have revealed the complete PAL-derived pathway for SA biosynthesis in rice, including its cellular compartmentalization (Fig. 5d). Our findings provide critical insight into the core SA biosynthetic machinery in plants, especially in staple crops, and identify promising genetic targets for engineering disease resistance through SA pathway modulation.

## Methods

### Plant materials and growth conditions

The rice (*O. sativa* L.) varieties Zhonghua 11 (ZH11), Xiushui 11 (XS11), IRBB7, Wuyujing 3 (WYJ3), IR64 and Wuyunjing 8 (WYJ8) were used as wild type. Tobacco (*N. tabacum* cv. *Samsun*-*NN*) and maize (*Zea mays* cv. B104) were used as WT. *A. thaliana*, *B. napus*, *Glycine max, Pinus elliottii*, *Ginkgo biloba*, *Capsicum annuum*, *Solanum melongena, Populus canadensis, Chlamydomonas reinhardtii*, *Marchantia polymorpha*, *Sphagnum palustre*, *Selaginella moellendorffii*, *Nymphaea colorata* and *Salix babylonica* were collected from growth chambers or the field for isotope-labelling feeding experiments. The plants were grown in the growth chamber with a 12-h light (28 °C):12-h dark (22 °C) photoperiod, 500–600 μmol m^−2^ s^−1^ light intensity and 50% humidity. In the field experiments, the plants were grown under a conventional cultivation environment in a paddy field of the Botany Garden of Zhejiang Normal University in Jinhua (119° 63′ E, 29° 130′ N), China.

### EMS mutagenesis and forward genetic screen

The seeds of wild-type plants were mutagenized by EMS as described^47^. The mutants were screened from the M2 seedlings following a modified method based on a bacterial biosensor, *Acinetobacter* sp. ADPWH_*lux*^36,37^. In brief, ~0.05 g young leaves of 3-week plants were collected and placed into a well of 2-ml 96-well plates containing 600 μl LB. Then the samples were incubated in the water bath at 95 °C for 30 min. After the samples were cooled to room temperature, 50 μl of leaf extract was successfully transferred to a new black 96-well cell culture plate, and 50-μl culture of the biosensor strain *Acinetobacter* sp. ADPWH_*lux* (OD_600_ = 0.4) was added and mixed. The plates were incubated at 37 °C for 90 min, and the luminescence was read using Infinite 2000 PRO (Tecan).

### Map-based cloning and bulk population sequencing

F_1_ generation plants were obtained by crossing *osd1-1* with IR64 rice variety. All individual plants with low SA content in the F_2_ population were selected for DNA extraction. The simple sequence repeats (SSR) and sequence-tagged-site (STS) markers were screened for polymorphic markers. The PCR products were separated by 5% agarose gel electrophoresis. All the primers used in this study are listed in Supplementary Table 3.

For bulk population sequencing, an equal amount of DNA was extracted from four DNA pools, the *osd1-1*-type pool (44 lines) and the WT-type pool (50 lines) randomly selected from the BC_1_F_2_ individuals of *osd1-1* and XS11, the parent WT pool (50 lines), and the *osd1-1* mutant pool (50 lines). The library was prepared with the Illumina TruSeq DNA PCR-free prep kit and sequenced using the Illumina HiSeq X-ten platform. To identify the mutation site, we mapped the reads to the rice reference genome using BWA-MEM (v.0.7.17) with the default parameters. Alignments were sorted with SAM tools (v.1.6) and duplicates were marked with Picard Tools (v.2.27.5+dfsg). SNPs were called with SAM tools (v.1.6)/BCF tools (v.1.5)^48^. To reduce false-positive detection of SNPs, SNP positions with a SNP quality score. In brief, the ratio between the number of reads of a mutant SNP and the total number of reads covering the SNP site was defined as the SNP index. The ΔSNP index is defined by subtracting the SNP index value of the WT-type pool from the *osd1-1*-type pool. The average of ΔSNP index was calculated using a sliding-window approach with a 25-kb window size and a step size of 5 kb, and plotted across the 12 rice chromosomes.

### Quantification of SA, SAG, BB and BS in plants

SA and SAG in plants were extracted and quantified as described^49^. SA, SAG, BB and BS contents shown in Extended Data Fig. 8a–c were extracted as described^49^ with some modification. In brief, ~50 mg of leaf tissue was collected and flash frozen in liquid nitrogen, finely ground with freezing grinder and extracted with 500 μl methanol with internal standards (D_6_-SA, D-1156, C/D/N ISOTopes) at 4 °C for 4 h. After centrifugation, 150 μl supernatant was taken out for quantification of BB and BS by the high-resolution gas chromatography–mass spectrometry system (HRGC–MS, Thermo Fisher Scientific) which consisted of a Trace1610 series GC, an AS 1610 Liquid Autosampler and an Exactive GC Orbitrap MS analyser with electron ionization. The remaining mixture was subsequently extracted twice with 1 ml 80% methanol and 500-μl 100% methanol at 4 °C for 4 h. After centrifugation, the supernatant was collected and dried by nitrogen gas. Then the residue was resolved in 300 μl of 30% methanol for quantification of SA and SAG by the ExionLC (AB SCIEX) high-performance liquid chromatography (HPLC) instrument paired with a QTRAP 5500 mass spectrometer (AB SCIEX).

### CRISPR–Cas9 gene editing and gene overexpression

The *osd2* and *osd3* mutants in the ZH11 background were obtained from BIOGLE GeneTech (http://biogle.cn). The *osd4* mutants in XS11 background and *oskat1 kat2* double mutants in ZH11 background were generated by CRISPR–Cas9 genome editing technology as described^50^. The tobacco *nsd1* and *nsd3* mutants were generated by CRISPR–Cas9 genome editing technology as described^51,52^. The maize *zsd3* double mutants were generated from wild-type B104 by Wuhan EDGENE Biotechnology. The mutation of CRISPR-mediated mutants was confirmed by DNA sequencing.

The coding sequences (CDS) of *OSD1*, Os*KAT2*, *OSD2*, *OSD3* and *OSD4* genes were amplified by PCR and cloned into pCR8 (K250020, Thermo Fisher Scientific). Then the constructed entry vectors were cloned into the binary vector pUbi-pMDC32 or pMDC43 to construct pUbi::OSD1, 35S::GFP-OsKAT2, pUbi::OSD2, 35S::GFP-OSD3, and 35S::GFP-OSD4 vectors by the Gateway LR Clonase II enzyme mix (Thermo Fisher Scientific, USA) or ClonExpress II One Step Cloning Kit (Vazyme Biotech, China), respectively. Binary vectors were transformed into rice by *Agrobacterium tumefaciens*-mediated transformation.

### Pathogen test and trypan blue staining

Rice plants grown in paddy fields were inoculated with *X. oryzae* pv. *oryzae* (*Xoo*) Philippine strain P6 (*PXO99*^*A*^) at the tillering stage following a leaf-clipping method as described^53,54^. *Xoo* was cultured on agar medium that contained 20 g sucrose, 5 g peptone, 0.5 g Ca(NO_3_)_2_, 0.43 g Na_2_HPO_4_ and 0.05 g FeSO_4_ per litre and were cultured at 28 °C for 2–3 days. The culture was resuspended with sterile water to the optical density at 600 nm (OD_600_) = 1.0 and immediately used for plant inoculation. The infected symptoms were photographed and measured at 14 days post-inoculation.

The fully expanded leaves from rice plants at the tillering stages were used for syringe infiltration to observe the hypersensitive reaction (HR) following a described method^55^. In brief, bacterial suspensions of optical density of OD_600_ = 0.5 were used for syringe infiltration. The plants were grown in the growth chamber with a 12-h light (30 °C):12-h dark (28 °C) photoperiod, 500–600 μmol m^−2^ s^−1^ light intensity, and 80% humidity 7 days before infiltration. Samples were taken before and 48 h after infiltration to analyse the content of SA, SAG, BB and BS. Trypan blue staining of the HR reactions was performed 3 dpi following a previously described method^56^. The phenotype of the HR reactions was observed at 4 dpi. The images were captured by using a stereomicroscope (SteREO Discovery.V12, Carl Zeiss Microscopy).

### Transcriptional analysis of gene expression

Total RNAs were extracted with the Trizol reagent (Aidlab) from different tissues of the 70-day-old ZH11 plants. The 4-cm leaf truncation below the cut edge was collected at 12, 24, 48 and 72 h post-inoculation of *Xoo*. RT–qPCR was performed using SYBR Green (Q712, Vazyme Biotech) on the QuantStudio 1 Real-Time PCR Thermal Cycler (Thermo Fisher Scientific) according to the manufacturer’s instructions.

### Protein subcellular localization

To generate pCR8-AIM1 and pCR8-OsKAT1, the CDS of *AIM1* and Os*KAT1* were amplified and inserted into pCR8. Then, the constructed entry vectors of *OSD1*–*4*, Os*KAT1*/*KAT2* were cloned into the binary vector pSAT6 or pMDC43 to construct GFP fusion expression vectors. The pCR8-AIM1 was cloned into the binary vector mCherry-pSAT6 to construct 35S::mCherry-*Os*AIM1 vector as a peroxisome-localized marker.

The rice protoplasts were prepared and transformed with expression vectors following a previously described method with some modification^57^. In brief, the stem and sheath tissues from rice seedlings (2 to 3 weeks old) were cut into approximately 0.5 mm strips. The strips were immediately transferred into 0.6 M mannitol for quick plasmolysis treatment, followed by enzymatic digestion in the dark with gentle shaking. The protoplasts were collected by filtration through 40 μm nylon meshes. After transfection with the vector by using the PEG-mediated transfection approach, the protoplasts were incubated at 22 °C for 10 h. The fluorescence images were observed by the Zeiss LSM 880 Confocal Microscope system (Carl Zeiss Microscopy) using an excitation 488-nm laser with an emission wavelength of 505–550 nm for GFP, a 561-nm laser with an emission wavelength of 600–660 nm for mCherry, and a 488-nm laser with an emission wavelength of 650–710 nm for chloroplast. mCherry–AIM1^23^ and mCherry–HDEL^41^ were used as peroxisome and endoplasmic reticulum-localized markers, respectively.

Endoplasmic reticulum membrane preparation was carried out as previously described^58^. Immunoblots were probed with antibodies against GFP (Invitrogen A6455, 1:3,000), *A. thaliana* fructose-1,6-bisphosphatase (PhytoAB, PHY3095A, 1:3,000), or *A. thaliana* heat shock 70 kDa protein BIP1/2 (PhytoAB, PHY1481A, 1:1,000), and goat anti-rabbit IgG antibody (PhytoAB, PHY6000, 1:1,000), according to standard procedures.

### Heterologous expression and purification of recombinant proteins

The CDSs of different genes were amplified by PCR using gene-specific primers from rice cDNAs, and cloned into pMAL-c2X (New England BioLabs). The Ph*CHD* gene was synthesized and cloned into pMAL-c5X (New England BioLabs) by Beijing Tsingke Biotech. The correctly sequenced plasmids were transformed into *E. coli* BL21 (DE3, pLys3; Invitrogen) or Rosetta 2 (DE3, Beyotime). The culture was induced for protein expression with 0.6 mM IPTG, and then incubated for 24 h at 18 °C. The recombinant protein was purified according to the manufacturer’s protocol of Amylose Resin (New England BioLabs).

The CDS of *OSD3* was amplified from the pCR8-OSD3 and cloned into pESC-URA vector (Agilent Technologies) to generate Flag–OSD3 fusion protein expression vector using Clonexpress II One Step Cloning Kit. The plasmid was transformed into yeast (*S. cerevisiae*) strain WAT11. Yeast cells carrying pESC-URA or pESC-URA-OSD3 were cultured in 5 l of medium and induced by 2% galactose. Microsomal proteins were purified according to a previously described method^59^. The protein concentration was determined by the Bradford assay^60^. The immunoblots were probed with antibody against Flag (F3165, Sigma-Aldrich, 1:1,000), and goat anti-mouse IgG antibody (BS12478, Bioworld, 1:1,000), according to standard procedures.

### In vitro biochemical enzyme assays

The OSD1 enzyme assay was performed as described^30,38^. The reaction mixture contained 100 mM Tris-HCl buffer (pH 7.5), 2.5 mM MgCl_2_, 1 mM ATP (D7378, Beyotime), 0.8 mM CoA (ST353, Beyotime), 0.3 mM *trans*-CA and around 0.45 μg μl^−1^ MBP–OSD1 protein. The mixture was incubated at 25 °C for 30 min and then terminated by boiling for 3 min. The enzyme activity was calculated by using the *trans*-CA consumption rate.

The AIM1 enzyme activities were assayed following a method with modification^25^. The reaction mixture contained 100 mM Tris-HCl buffer (pH 7.5), 2.5 mM MgCl_2_, 50 mM KCl, 0.5 mM CA-CoA, 1 mM pyruvic acid, 1 mM NAD^+^, 2 units of lactate dehydrogenase, and 0.185 μg μl^−1^ MBP–AIM1 protein. The mixture was incubated at 30 °C for 30 min and terminated by boiling for 3 min. The MBP–PhCHD enzyme was used as a positive control^25^.

The conditions for AIM1 and *Os*KAT coupling enzyme assays were developed according to the literature^25,61^. The reaction mixture contained 100 mM Tris-HCl buffer (pH 7.5), 2.5 mM MgCl_2_, 50 mM KCl, 0.5 mM CA-CoA, 2 mM CoA, 1 mM pyruvic acid, 1 mM NAD^+^, 2 units of lactate dehydrogenase, 0.075 μg μl^−1^ AIM1 protein and 0.112 μg μl^−1^ *Os*KAT1 protein or 0.065 μg μl^−1^ *Os*KAT2 protein. The mixture was incubated at 30 °C for 20 min and terminated by boiling for 3 min.

The enzyme assay of OSD2 was developed according to the literature^44,45^. The reaction mixture contained 200 mM phosphate buffer (pH 6.0), 300 mM NaCl, 1 mM dithiothreitol, 0.4 mM BA-CoA, 0.5 mM BAlc (Boer) and 0.25 μg μl^−1^ OSD2 protein. The mixture was incubated at 10 °C for 30 min. All reactions were stopped by adding an equal volume of 100% methanol to denature the protein. After centrifugation at 13,000*g* for 10 min, the supernatant was air-dried with nitrogen gas and then resuspended with 300 μl methanol.

The OSD3 activities were assayed essentially as described previously^62^, with some modifications. The reaction mixture (500 μl) containing 80 mM sodium phosphate (pH 7.0), 5 mM DTT, 5 mM NADPH (ST360, Beyotime), 50 μM BB (RHAWN), and 2.5 μg μl^−1^ microsomal proteins was incubated at 30 °C for 1 h. All the reaction was terminated by the addition of 100 μl acetic acid. The mixed solution was mixed with 1 ml water statured ethyl acetate, and centrifuged at 13,000*g* for 10 min.

The enzyme assay of OSD4 was developed according to the literature^63^. The reaction mixture contained 100 mM Tris-HCl buffer (pH 8.0), 0.6 mM BS (Aladdin), and 0.48 μg μl^−1^ OSD4 protein. The mixture was incubated at 30 °C for 30 min. The reaction was stopped with an equal volume of acetonitrile.

For substrate specificities of the enzymes, the compounds from related biosynthetic pathways or structurally similar substrates were tested under optimal reaction conditions. For kinetic analysis, an appropriate enzyme concentration and incubation time were chosen. All the supernatants of enzyme reaction products were filtered by 0.22-μm membrane filter and analysed by LC–MS, LC–UV–MS or GC–MS. The enzyme kinetic parameters were determined by the Michaelis–Menten equation for OSD1 and OSD4 or the allosteric sigmoidal enzyme kinetics equation for OSD2 by using GraphPad Prism (v.9.3.1).

### LC–MS and LC–UV–MS analysis

The chromatographic peak retention time and DAD spectrum of enzymatic products were analysed by the ExionLC (AB SCIEX), which consisted of a controller, an AD autosampler, two AD pumps, an AD column oven, and a photo-diode array detector. Detailed conditions are shown in Supplementary Table 4. The mass spectra of the enzymatic products of OSD1, AIM1 and AIM1 coupling with *Os*KAT1/KAT2, OSD3 and OSD4 were characterized by a TripleTOF 4600 mass analyser (AB SCIEX) paired with the Nexera X2 HPLC System (Shimadzu). The TripleTOF 4600 mass analyser was equipped with electrospray ionization (ESI). The Nexera X2 HPLC instrument consisted of a DGU-20A degasser, a SIL-30AC autosampler, two LC-30AD pumps, a CTO-20AC column Oven, and an SPD-20A detector. Detailed conditions are shown in Supplementary Tables 5 and 6.

The SA and its derivatives were quantified by the ExionLC (AB SCIEX) paired with a QTRAP 5500 mass spectrometer (AB SCIEX). The QTRAP 5500 mass spectrometer was equipped with an electrospray ionization interface (ESI, Turbo V). The multiple reaction monitoring (MRM) mode was used, and the mobile phase, flow programme, and the specific precursor ion-to-product ion transitions of all target compounds with the detailed conditions were described in Supplementary Table 7. The SA and SAG were accurately quantified using internal standards, and the concentration of other compounds was calculated according to the standard curve of the standards. Both data acquisition and instrument control were coordinated by Analyst Software (v.1.6.3).

### GC–MS analysis

The GC–MS system (a 7890B GC coupled with 5977B mass spectrometer detector, Agilent Technologies) was used for BB content quantification in OSD2 enzyme assay. The detailed conditions are shown in Supplementary Table 8, and the concentration of BB was quantified according to the standard curve.

The HRGC–MS system (a Trace1610 series GC coupled to an Exactive GC Orbitrap mass analyser, Thermo Fisher) was used to characterize the mass spectra of OSD2 enzymatic products and quantify the BB and BS in plant samples according to the standard curve. The detailed conditions are shown in Supplementary Table 8.

### Inference of homologues of key components in PAL-SA pathway

To identify the closely related homologues of key components in PAL-SA pathway of rice, a total of 25 plant species with high-quality genomes from representative taxonomic groups (Rhodophyta, Chlorophyta, Streptophyte algae, Charophyta, Bryophyta, Lycophyta, Monilophyta, Gymnospermae, Basal angiosperms, Monocots and Eudicots) in the plant kingdom were downloaded from public database, including EnsemblPlants, FigShare, FernBase, GinkgoDB, Nicomics, ORCAE, Phytozome 13 and TreeGenes (Supplementary Table 9). Whole protein sequences from the above 25 species genomes with the longest transcripts were retained as representative isoforms. To obtain high-quality protein sequences, we removed the possibly misannotated peptides with starting amino acids other than methionine and sequences containing unknown amino acid X using an in-house script. Then, STRIDE was used to infer the species tree based on the identified orthogroups^64^. For nodes in inferred species with low support rate (bootstrap values < 90), we correct the phylogenetic relationship among these species according to the related literatures^65,66^. Based on the corrected species, the closely related homologues of these key components (OSD1, AIM1, *Os*KAT1, *Os*KAT2, OSD2, OSD3 and OSD4) were identified using Orthofinder 2.5.5^67^. The conserved protein PFAM domains for these putative homologues were identified by InterProScan (v.5.69-101.0)^68^, PF00501 and PF13193 for *OSD1* (*Os03g0130100*), PF00378, PF02737 and PF00725 for *AIM1* (*Os02g0274100*), PF00108 and PF02803 for Os*KAT1* (*Os02g0817700*) and Os*KAT2* (*Os10g0457600*), PF02458 for *OSD2* (*Os10g0503300*), PF00067 for *OSD3* (*Os09g0441400*) and PF07859 for *OSD4* (*Os05g0410200*), respectively. The retained protein sequences with at least one conserved domain (Supplementary Table 10) were then used for multiple sequence alignment with MAFFT v7.526^69^ and construction of maximum-likelihood gene trees with 500 bootstrap replicates and optimal model using RAxML (v.8.2.12)^70^. Final gene trees for each component were constructed after removing protein sequences with extremely long branch (Extended Data Fig. 9). The retained protein sequence sets were considered as the closely related homologues of these PAL-SA pathway enzymes in rice (Supplementary Table 11).

### Substrate/intermediate feeding and isotope-labelling tracer experiments

For substrate/intermediate feeding, 200 μM CA, BB, BS, or SA were separately prepared in a water solution containing 0.1% Tween-20. The WT and SA-deficient mutants at the tillering stage were applied foliar spray with the solution twice a day (for six days) before further analysis.

The isotope-labelling tracer experiments were followed a previously described method with modification^46^. Leaves from different plants were cut and incubated in water with 200 μM ring-^13^C-labelled phenylalanine (^13^C_6_-Phe, Cambridge Isotope Laboratories) for 72 h. SA, SAG, ^13^C_6_-SA, and ^13^C_6_-SAG were extracted from the samples and analysed as described previously^49^.

### Statistical analysis

Statistical significance was determined using two-sided Student’s *t*-tests or one-way ANOVA with LSD test for multiple groups (≥3) of data. Statistical analysis was performed using GraphPad Prism 9.3 or IBM SPSS Statistics 21. Detailed statistical analyses are explained in the figure legends, and *P* values are indicated in the figures or the source data.

### Reporting summary

Further information on research design is available in the Nature Portfolio Reporting Summary linked to this article.

## Online content

Any methods, additional references, Nature Portfolio reporting summaries, source data, extended data, supplementary information, acknowledgements, peer review information; details of author contributions and competing interests; and statements of data and code availability are available at 10.1038/s41586-025-09175-9.

## Supplementary information

Supplementary Information Supplementary Fig. 1: original source images for western blots.

Reporting Summary

Supplementary Table 1: The top 50 co-expressed genes of *Os03g0130100* (*OSD1*).

Supplementary Table 2: Exact *P* values in Figs. 2b and 3b.

Supplementary Table 3: List of primers used in this study.

Supplementary Table 4: HPLC parameters for testing the chromatography and DAD spectrum of enzyme product.

Supplementary Table 5: HPLC and MS parameters for testing the mass spectra of enzyme product.

Supplementary Table 6: HPLC and MS parameters for testing the MS/MS spectra of enzyme product.

Supplementary Table 7: MRM acquisition settings for the selected metabolites.

Supplementary Table 8: The parameters for GC–MS.

Supplementary Table 9: The websites for downloading the 25 related plant genomes.

Supplementary Table 10: The retained protein sequences used for multiple sequence alignment and construction of maximum likelihood gene trees.

Supplementary Table 11: The retained protein sequence sets used as the closely related homologues of these PAL-derived SA biosynthetic pathway enzymes in rice.

## Source data

Source Data Fig. 1

Source Data Fig. 2

Source Data Fig. 3

Source Data Fig. 4

Source Data Fig. 5

Source Data Extended Data Fig. 1

Source Data Extended Data Fig. 2

Source Data Extended Data Fig. 5

Source Data Extended Data Fig. 6

Source Data Extended Data Fig. 7

Source Data Extended Data Fig. 8

Source Data Extended Data Fig. 10

## Data Availability

All the data generated in this study are available in the paper and the Supplementary Information. Rice sequence data from this article are available from the National Center for Biotechnology Information (NCBI) website (https://www.ncbi.nlm.nih.gov/, BioProject, PRJNA13139) and rice genome annotation project website (https://www.ricedata.cn/gene/) under the following accessions: *OSD1* (*Os03g0130100*), *AIM1* (*Os02g0274100*), Os*KAT1* (*Os02g0817700*), Os*KAT2* (*Os10g0457600*), *OSD2* (*Os10g0503300*), *OSD3* (*Os09g0441400*), *OSD4* (*Os05g0410200*) and Os*UBQ5* (*Os01g0328400*). Tobacco sequence data from this article are available from the NCBI (BioProject, PRJNA208209) under accessions *NSD1-a* (*LOC107815113*), *NSD1-b* (*LOC107761717*), *NSD1-c* (*LOC107770426*), *NSD1-d* (*LOC107783557*), *NSD3-a* (*LOC107803700*), *NSD3-b* (*LOC107823191*), *NSD3-c* (*LOC107823192*) and *NSD3-d* (*LOC107803699*). *Z. mays* sequence data from this article are available from the NCBI (BioProject, PRJNA10769) under accessions *ZSD3-1* (*Zm00001d005823*) and *ZSD3-2* (*Zm00001d020628*). The complete protein sequences of the species mentioned in this study are available from EnsemblPlants, FigShare, FernBase, GinkgoDB, Nicomics, ORCAE, Phytozome 13 and TreeGenes, and the download link for each species can be found in Supplementary Table 9. Uncropped gel and immunoblotting images are provided in Supplementary Fig. 1. Source data are provided with this paper.
