# Supplementary material for: Complete biosynthesis of salicylic acid from phenylalanine in plants
Source: Nature. 2025 Jul 23;645(8079):218–27. doi: 10.1038/s41586-025-09175-9 (PMC12408352; doi:10.1038/s41586-025-09175-9)
Supplement: Supplementary file 1 — Supplementary Fig. 1: original source images for western blots. [file 41586_2025_9175_MOESM1_ESM.pdf]

nature portfolio

https://doi.org/10.1038/s41586-025-09175-9

Supplementary information

Complete biosynthesis of salicylic acid from phenylalanine in plants

In the format provided by the
authors and unedited

Extended Data Fig. 6 a

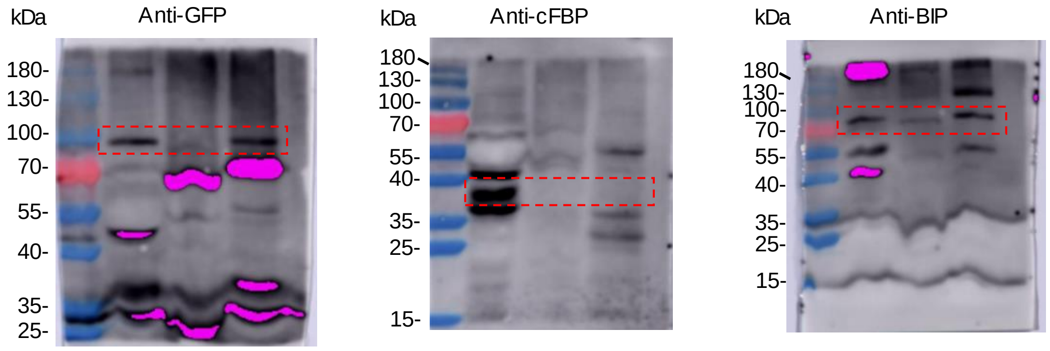

Extended Data Fig. 6 b

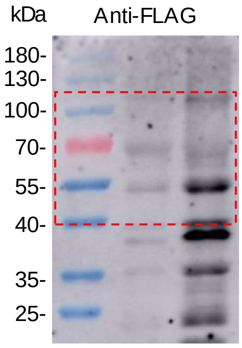

Supplementary Figure 1. The original source images for western blots.
