# Supplementary material for: Complete biosynthesis of salicylic acid from phenylalanine in plants
Source: Nature. 2025 Jul 23;645(8079):218–27. doi: 10.1038/s41586-025-09175-9 (PMC12408352; doi:10.1038/s41586-025-09175-9)
Supplement: Supplementary file 2 — Reporting Summary [file 41586_2025_9175_MOESM2_ESM.pdf]

nature portfolio

Corresponding author(s): Kewei Zhang; Yanjun Zhang; Chang-Jun Liu

Last updated by author(s): May 6, 2025

Reporting Summary

Nature Portfolio wishes to improve the reproducibility of the work that we publish. This form provides structure for consistency and transparency in reporting. For further information on Nature Portfolio policies, see our Editorial Policies and the Editorial Policy Checklist.

Statistics

For all statistical analyses, confirm that the following items are present in the figure legend, table legend, main text, or Methods section.

n/a	Confirmed	
☐	☒	The exact sample size (*n*) for each experimental group/condition, given as a discrete number and unit of measurement
☐	☒	A statement on whether measurements were taken from distinct samples or whether the same sample was measured repeatedly
☐	☒	The statistical test(s) used AND whether they are one- or two-sided<br>*Only common tests should be described solely by name; describe more complex techniques in the Methods section.*
☒	☐	A description of all covariates tested
☐	☒	A description of any assumptions or corrections, such as tests of normality and adjustment for multiple comparisons
☐	☒	A full description of the statistical parameters including central tendency (e.g. means) or other basic estimates (e.g. regression coefficient) AND variation (e.g. standard deviation) or associated estimates of uncertainty (e.g. confidence intervals)
☐	☒	For null hypothesis testing, the test statistic (e.g. *F*, *t*, *r*) with confidence intervals, effect sizes, degrees of freedom and *P* value noted<br>*Give P values as exact values whenever suitable.*
☒	☐	For Bayesian analysis, information on the choice of priors and Markov chain Monte Carlo settings
☒	☐	For hierarchical and complex designs, identification of the appropriate level for tests and full reporting of outcomes
☒	☐	Estimates of effect sizes (e.g. Cohen's *d*, Pearson's *r*), indicating how they were calculated

Our web collection on statistics for biologists contains articles on many of the points above.

Software and code

Policy information about availability of computer code

Data collection

Confocal microscopy: Zeiss LSM 880
Gene expression （RT-qPCR): Thermo Fisher Scientific （QuantStudio 1）
Western blots: Amersham Imagine 600
GC-MS: a 5977B mass spectrometer detector (Agilent Technologies) combined with 7890B GC (Agilent Technologies) with an Agilent 19091S-433 capillary column (HP-5MS, 30 m*250 µm*0.25 µm). Both data acquisition and instrument control were coordinated by MassHunter GC/MS acquisition (version B07.06.2704)
HRGC-MS：an Exactive GC-Orbitrap MS combined with a Trace1610 series GC (Thermo Fisher Scientific) with TG-5SILMS columna (30 m*250 µm*0.25 µm). Both data acquisition and instrument control were coordinated by Thermo Scientific Xcalibur (version 4.7.69.37)
LC-MS: the ExionLC (AB SCIEX) high-performance liquid chromatography (HPLC) instrument paired with a QTRAP 5500 mass spectrometer (AB SCIEX). The ExionLC instrument consisted of a controller, an AD autosampler, two AD pumps, an AD column Oven, and a photo-diode array detector (PDA). The QTRAP 5500 mass spectrometer was equipped with an electrospray ionization interface (ESI, Turbo V). The Acquity UPLC BEH C18 column (2.1*100 mm, particle size of 1.7 µm) and Acquity UPLC CSH C18 column (2.1*150 mm, particle size of 1.7 µm) were used. Both data acquisition and instrument control were coordinated by Analyst Software (version 1.6.3)
LC-MS: a TripleTOF 4600 mass analyzer (AB SCIEX) paired with the Nexera X2 HPLC System (SHIMADZU). The TripleTOF 4600 mass analyzer was equipped with electrospray ionization (ESI). The Nexera X2 HPLC instrument consisted of a DGU-20A degasser, a SIL-30AC autosampler, two LC-30AD pumps, a CTO-20AC column Oven, and an SPD-20A detector. Acquity UPLC CSH C18 column (2.1*150 mm, particle size of 1.7 µm) was used. Both data acquisition and instrument control were coordinated by Analyst TF Software (version 1.7)
Photographing of plants: Canon camera 60D
Bioinformatics analysis: a total of 25 plant species with high-quality genomes from representative taxonomic groups (Rhodophyta, Chlorophyta, Streptophyte algae, Charophyta, Bryophyta, Lycophyta, Monilophyta, Gymnospermae, Basal angiosperms, Monocots, and Eudicots) in plant kingdom were downloaded from public database, including EnsemblPlants, FigShare, FernBase, GinkgoDB, Nicomics, ORCAE,

Phytozome 13, and TreeGenes.
HR phenotypes: stereomicroscope (SteREO Discovery.V12, Carl Zeiss Microscopy GmbH, Jena, Germany).

Data analysis

Confocal images and HR phenotypes were analyzed with Zen 2.3 (Blue edition).
Graphics drawing, statistical analysis by GraphPad Prism software (version 9.3).
Multiple sequence alignment was performed by Snapgene (version 3.2.1).
GC/MS: Mass Hunter workstation software qualitative analysis navigator (version B.08.00).
HRGC-MS：Thermo Scientific Xcalibur (version 4.7.69.37)
LC/MS: Analyst Software (version 1.6.3) or Analyst TF Software (version 1.7).
Bioinformatics analysis: To identify the closely related homologs of key components in PAL-SA pathway of rice, a total of 25 plant species with high-quality genomes from representative taxonomic groups (Rhodophyta, Chlorophyta, Streptophyte algae, Charophyta, Bryophyta, Lycophyta, Monilophyta, Gymnospermae, Basal angiosperms, Monocots, and Eudicots) in plant kingdom were downloaded from public database, including EnsemblPlants, FigShare, FernBase, GinkgoDB, Nicomics, ORCAE, Phytozome 13, and TreeGenes (Supplementary Table 8). Whole protein sequences from the above 25 species genomes with the longest transcripts were retained as representative isoforms. To obtain high-quality protein sequences, we removed the possibly misannotated peptides with starting amino acids other than methionine and sequences containing unknown amino acid "X" using in-house script. Then, STRIDE was used to infer the species tree based on the identified orthogroups. For nodes in inferred species with low support rate (bootstrap values < 90), we correct the phylogenetic relationship among these species according to the related literatures. Based on the corrected species, the closely related homologs of these key components (OSD1, AIM1, OsKAT1, OsKAT2, OSD2, OSD3, and OSD4) were identified using Orthofinder 2.5.5. The conserved protein PFAM domains for these putative homologs were identified by InterProScan (version 5.69-101.0), PF00501 and PF13193 for OSD1 (Os03g0130100), PF00378, PF02737, and PF00725 for AIM1 (Os02g0274100), PF00108 and PF02803 for OsKAT1 (Os02g0817700) and OsKAT2 (Os10g0457600), PF02458 for OSD2 (Os10g0503300), PF00067 for OSD3 (Os09g0441400), and PF07859 for OSD4 (Os05g0410200), respectively. The retained protein sequences with at least one conserved domain (Supplementary Table 9) were then used for multiple sequence alignment with MAFFT v7.526 and construction of maximum likelihood gene trees with 500 bootstrap replicates and optimal model using RAxML (version 8.2.12). Final gene trees for each component were constructed after removing protein sequences with extremely long branch (Extended Data Fig. 9). The retained protein sequence sets were considered as the closely related homologs of these PAL-SA pathway enzymes in rice (Supplementary Table 10).
One-way ANOVA analysis was used IBM SPSS Statistics 21.
Bulk population sequencing: To identify the mutation site, we mapped the reads to the rice reference genome using BWA-MEM (version 0.7.17) with the default parameters. Alignments were sorted with SAM tools (version 1.6) and duplicates were marked with Picard Tools (version 2.27.5+dfsg). SNPs were called with SAM tools (version 1.6)/BCF tools (version 1.5).

For manuscripts utilizing custom algorithms or software that are central to the research but not yet described in published literature, software must be made available to editors and reviewers. We strongly encourage code deposition in a community repository (e.g. GitHub). See the Nature Portfolio guidelines for submitting code & software for further information.

Data

Policy information about availability of data

All manuscripts must include a data availability statement. This statement should provide the following information, where applicable:

- Accession codes, unique identifiers, or web links for publicly available datasets
- A description of any restrictions on data availability
- For clinical datasets or third party data, please ensure that the statement adheres to our policy

All the data generated in this study are available in the paper and the Supplementary Information. All the materials needed to replicate the work are available. Rice sequence data from this article are available from the National Center for Biotechnology Information (NCBI) website (https://www.ncbi.nlm.nih.gov/) and rice genome annotation project website (https://www.ricedata.cn/gene/) by the following accession number: OSD1 (Os03g0130100), AIM1 (Os02g0274100), OsKAT1 (Os02g0817700), OsKAT2 (Os10g0457600), OSD2 (Os10g0503300), OSD3 (Os09g0441400), OSD4 (Os05g0410200), OsUBQ5 (Os01g0328400). Tabacco sequence data from this article are available from the NCBI by the following accession number: NSD1-a (LOC107815113), NSD1-b (LOC107761717), NSD1-c (LOC107770426), NSD1-d (LOC107783557), NSD3-a (LOC107803700), NSD3-b (LOC107823191), NSD3-c (LOC107823192), NSD3-d (LOC107803699). Zea mays sequence data from this article are available from the NCBI by the following accession number: ZSD3-1 (Zm00001d005823), ZSD3-2 (Zm00001d020628). The complete protein sequences of the species mentioned in this study are available from the following databases, EnsemblPlants, FigShare, FernBase, GinkgoDB, Nicomics, ORCAE, Phytozome 13, and TreeGenes, and the download link for each species can be found in Supplementary Table 8. Uncropped gel and immunoblotting images are provided in Supplementary Fig. 1. Source data are provided with this paper.

Research involving human participants, their data, or biological material

Policy information about studies with human participants or human data. See also policy information about sex, gender (identity/presentation), and sexual orientation and race, ethnicity and racism.

Reporting on sex and gender	NA
Reporting on race, ethnicity, or other socially relevant groupings	NA
Population characteristics	NA
Recruitment	NA

Ethics oversight	NA

Note that full information on the approval of the study protocol must also be provided in the manuscript.

Field-specific reporting

Please select the one below that is the best fit for your research. If you are not sure, read the appropriate sections before making your selection.

☒ Life sciences ☐ Behavioural & social sciences ☐ Ecological, evolutionary & environmental sciences

For a reference copy of the document with all sections, see nature.com/documents/nr-reporting-summary-flat.pdf

Life sciences study design

All studies must disclose on these points even when the disclosure is negative.

Sample size	The sample size and the results of the statistical analysis are described in the relevant figures or method section. Sample size was based on experiments trials and previous publications on similar experiments. Xoo inoculation, quantification of SA, SAG, BB, BS, and biochemical assays of the enzymes in vitro: DOI: 10.1111/pce.14328; gene tissue/organ-specific expression pattern and agronomic traits: DOI 10.1093/plphys/kiac401; and pathogen induction gene expression: https://doi.org/10.1016/j.xplc.2021.100143.
Data exclusions	No data were excluded from analyses in the experiments.
Replication	All experiments were independently conducted at least twice with similar results. And the number of replicates is indicated in the figure legends.
Randomization	All samples were arranged randomly into experimental groups. Plants for experiments were grown side by side to minimize unexpected environmental variations during growth.
Blinding	Investigators were not blinded to the allocation in the experiments, which do not contain clinical trials. In addition, the research materials are gene edited plants, which need to be strictly regulated and clearly labeled during the experimental process, so the blinding design is not applicable to this system. Experiments were conducted by different authors, whenever possible.

Reporting for specific materials, systems and methods

We require information from authors about some types of materials, experimental systems and methods used in many studies. Here, indicate whether each material, system or method listed is relevant to your study. If you are not sure if a list item applies to your research, read the appropriate section before selecting a response.

Materials & experimental systems

n/a	Involved in the study	
☐	☒	Antibodies
☒	☐	Eukaryotic cell lines
☒	☐	Palaeontology and archaeology
☒	☐	Animals and other organisms
☒	☐	Clinical data
☒	☐	Dual use research of concern
☐	☒	Plants

Methods

n/a	Involved in the study	
☒	☐	ChIP-seq
☒	☐	Flow cytometry
☒	☐	MRI-based neuroimaging

Antibodies

Antibodies used	Anti Flag (F3165, Sigma-Aldrich), Goat anti mouse (BS12478, Bioworld),<br>Anit GFP (Invitrogen A6455), Anti-Cytosolic fructose-1,6 bisphosphatase (PhytoAB, PHY3095A), Arabidopsis heat shock 70 kDa protein BIP1/2 (PhytoAB, PHY1481A), Goat Anti-Rabbit (PhytoAB, PHY6000).
Validation	Anti Flag (F3165, Sigma-Aldrich): https://www.sigmaaldrich.cn/CN/zh/product/sigma/f3165<br>Goat anti mouse (BS12478, Bioworld): https://bioworlde.com/Secondary-Antibodies/124447.html<br>Anit GFP (Invitrogen A6455): https://www.thermofisher.cn/cn/zh/antibody/product/GFP-Antibody-Polyclonal/A-6455<br>Anti-Cytosolic fructose-1,6 bisphosphatase (PhytoAB, PHY3095A),:https://www.phytoab.com/cfbpase%20antibody<br>Arabidopsis heat shock 70 kDa protein BIP1/2 (PhytoAB, PHY1481A):https://www.phytoab.com/catalogsearch/result/?q=PHY1481A<br>Goat Anti-Rabbit (PhytoAB, PHY6000):https://www.phytoab.com/products/secondary-antibodies/goat-anti-rabbit-igg-h-l-hrp-1

Dual use research of concern

Policy information about dual use research of concern

Hazards

Could the accidental, deliberate or reckless misuse of agents or technologies generated in the work, or the application of information presented in the manuscript, pose a threat to:

No	Yes	
☒	☐	Public health
☒	☐	National security
☒	☐	Crops and/or livestock
☒	☐	Ecosystems
☒	☐	Any other significant area

Experiments of concern

Does the work involve any of these experiments of concern:

No	Yes	
☒	☐	Demonstrate how to render a vaccine ineffective
☒	☐	Confer resistance to therapeutically useful antibiotics or antiviral agents
☒	☐	Enhance the virulence of a pathogen or render a nonpathogen virulent
☒	☐	Increase transmissibility of a pathogen
☒	☐	Alter the host range of a pathogen
☒	☐	Enable evasion of diagnostic/detection modalities
☒	☐	Enable the weaponization of a biological agent or toxin
☒	☐	Any other potentially harmful combination of experiments and agents

Plants

Seed stocks	The rice (Oryza sativa) varieties Zhonghua 11 (ZH11), Xiushui 11 (XS11), IRBB7, Wuyujing 3 (WYJ3), IR64, and Wuyujing 8 (WYJ8) and Nicotiana tabacum cv. Samsum were stocked in our lab. The maize (Zea mays) variety B104 was purchased from Wuhan EDGENE Biotechnology (Wuhan, China).
Novel plant genotypes	The rice osd1 mutants were generated by EMS mutagenesis. The osd2 to 4 and oskat1 kat2 of rice, nsd1 and nsd3 of tobacco, and zsd3 of maize were all generated by CRISPR/Cas9 technology. The coding sequences of SA biosynthetic genes were cloned into the binary vector pUbi-pMDC32 or pMDC43 and were transformed into rice by Agrobacterium tumefaciens-mediated transformation.
Authentication	The mutations of the plants were verified by DNA sequencing. The gene expression levels of the over-expressed genes were quantified by RT-qPCR.
